# Role of MIF in coordinated expression of hepatic chemokines in patients with alcohol-associated hepatitis

**DOI:** 10.1172/jci.insight.141420

**Published:** 2021-06-08

**Authors:** Kyle L. Poulsen, Xiude Fan, Christopher D. Kibler, Emily Huang, Xiaoqin Wu, Megan R. McMullen, Lin Leng, Richard Bucala, Meritxell Ventura-Cots, Josepmaria Argemi, Ramon Bataller, Laura E. Nagy

**Affiliations:** 1 Center for Liver Disease Research, Department of Inflammation and Immunity, Cleveland Clinic, Cleveland, Ohio, USA.; 2 Department of Anesthesiology, McGovern Medical School, University of Texas Health Science Center at Houston, Houston, Texas, USA.; 3 Department of Infectious Diseases, First Affiliated Hospital of Xi’an Jiaotong University, Xi’an, Shaanxi, China.; 4 Department of Internal Medicine, Yale University School of Medicine, New Haven, Connecticut, USA.; 5 Division of Gastroenterology, Hepatology and Nutrition, University of Pittsburgh Medical Center, Pittsburgh Liver Research Center, Pittsburgh, Pennsylvania, USA.; 6 Gastroenterology and Hepatology, Cleveland Clinic, Cleveland, Ohio, USA.; 7 Department of Molecular Medicine, Case Western Reserve University, Cleveland, Ohio, USA.

**Keywords:** Hepatology, Inflammation, Chemokines, Hepatitis, Innate immunity

## Abstract

The chemokine system of ligands and receptors is implicated in the progression of alcohol-associated hepatitis (AH). Finding upstream regulators could lead to novel therapies. This study involved coordinated expression of chemokines in livers of healthy controls (HC) and patients with AH in 2 distinct cohorts of patients with various chronic liver diseases. Studies in cultured hepatocytes and in tissue-specific KO were used for mechanistic insight into a potential upstream regulator of chemokine expression in AH. Selected C-X-C chemokine members of the IL-8 chemokine family and C-C chemokine *CCL20* were highly associated with AH compared with HC but not in patients with liver diseases of other etiologies (nonalcoholic fatty liver disease [NAFLD] and hepatitis C virus [HCV]). Our previous studies implicate macrophage migration inhibitory factor (MIF) as a pleiotropic cytokine/chemokine with the potential to coordinately regulate chemokine expression in AH. LPS-stimulated expression of multiple chemokines in cultured hepatocytes was dependent on MIF. Gao-binge ethanol feeding to mice induced a similar coordinated chemokine expression in livers of WT mice; this was prevented in hepatocyte-specific *Mif*–KO (*Mif*^ΔHep^**) mice. This study demonstrates that patients with AH exhibit a specific, coordinately expressed chemokine signature and that hepatocyte-derived MIF might drive this inflammatory response.

## Introduction

Chemokines are small, leukocyte chemotactic proteins that are divided into families based upon conserved N-terminal cysteine (C) motifs. The C-C and C-X-C families are the most common, wherein the latter is classified C-X-C due to 2 cysteine residues being separated by any amino acid. The C-X-C family can be further subdivided into ELR^+^ and ELR^–^, named for an N-terminal tripeptide motif glutamate-leucine-arginine (ELR) adjacent to the C-X-C motif. An interesting feature of chemokine biology is the inherent redundancy in the system — e.g., multiple ligands for a given receptor and ligands can bind multiple receptors (1, 2). This biological redundancy of chemokine activity poses a significant challenge in targeting chemokine ligand–receptor interactions as potential therapeutics for the treatment of inflammatory diseases.

Chemokines are associated with the progression of many diseases, including alcohol-associated liver disease (ALD) (3–6). In rodent models of ALD, chemokines have been linked to progression of ethanol-induced liver injury. For example, CCL2, or monocyte chemoattractant protein-1 (MCP1), promotes monocyte infiltration, steatosis and liver injury following chronic ethanol feeding in mice (7) and CXCL1 is associated with liver injury via neutrophil accumulation in livers of mice fed a combination high-fat diet with acute ethanol binge (8). In patients with alcohol-associated hepatitis (AH), hepatic expression of the IL-8 family — which are all ELR^+^ C-X-C chemokines — was increased compared with healthy controls (HC) (4). Various studies have revealed that members of the IL-8 family (e.g., CXCL1, CXCL5, CXCL6, and CXCL8) are associated with liver-associated morbidity and patient mortality in patients with AH (4, 9–11). Furthermore, our group has identified a contributing role of macrophage migration inhibitory factor (MIF), a cytokine- and chemokine-like inflammatory mediator, in ALD via its control of hepatic inflammation and chemokine expression in mice. MIF is increased in liver and circulation of patients with AH and is associated with increased mortality in patients (12–14).

The goal of the current study was to evaluate whether the coordinated expression of the complex system of chemokines was altered in human and experimental ALD and to identify upstream cues that led to this phenomenon. We analyzed data from 2 different cohorts of patients. In the first cohort of patients with AH and HC, we compared expression of all genes in the liver and identified a chemokine signature highly associated with AH (GSE28619). Liver RNA sequencing (RNA-seq) data were analyzed from a second cohort of patients with different stages of ALD, from mild disease to severe AH, as were data from patients with nonalcoholic fatty liver disease (NAFLD) and hepatitis C virus (HCV). This second cohort allowed us to identify the distinct changes in chemokine expression that were specific to AH, as compared with other liver diseases. Finally, we wanted to assess what upstream signals might induce this coordinated expression program, and we investigated the role of the pleiotropic inflammatory factor MIF in controlling this specific program of chemokine expression. The hepatocyte has been implicated as an important source of MIF in ALD (13); therefore, we generated hepatocyte-specific MIF KOs to interrogate the role of hepatocyte-derived MIF in murine models of ethanol feeding. While changes in individual chemokine-receptor interactions have been studied in ALD, our analysis identified a potentially novel coordinated chemokine expression signature in the liver that is distinctive in patients with AH and likely dependent on MIF-mediated signaling.

## Results

### Gene network analysis identified a specific subset of chemokines associated with AH compared with HC.

**Figure 1:**
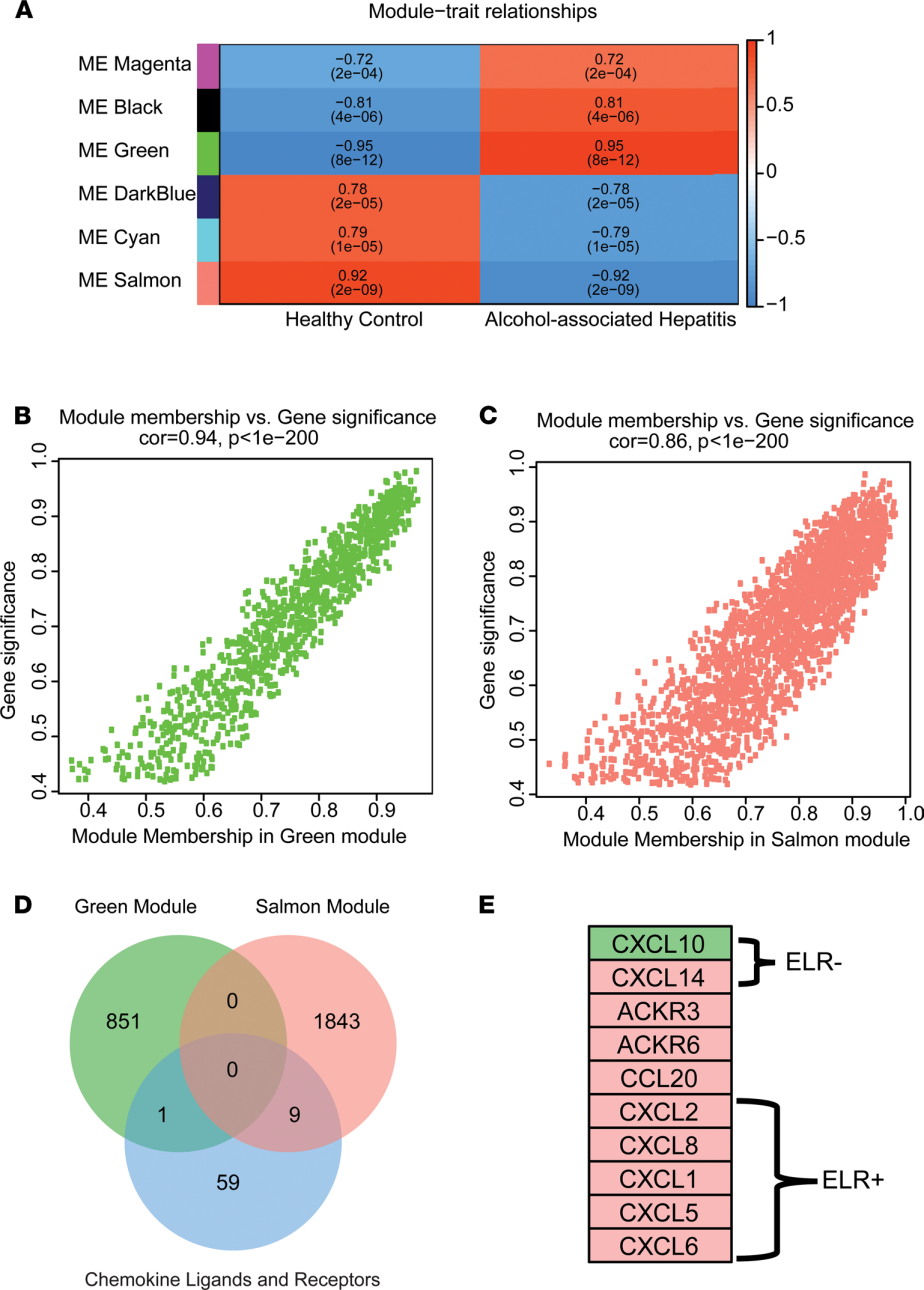
WGCNA identified specific gene modules related to alcohol-associated hepatitis. (**A**) The relationship of each color module to disease status. Correlation coefficients and *P* values are presented within each module per diagnosis. (**B** and **C**) Correlation of module membership versus gene significance was calculated in GSE28619 from green (**B**) and salmon (**C**) modules. (**D**) Venn diagram of the top 50% DEGs in the green module, salmon module, and chemokine ligands and receptors. (**E**) A total of 10 chemokine ligands and receptors were located in either the green or salmon modules.

To test the hypothesis that coordinated chemokine expression is evident in AH, we first analyzed publicly available microarray data that study gene expression on a whole transcriptomic level in patients with AH (11). Weighted gene correlation network analysis (WGCNA) was carried out to group interrelated gene expression patterns in livers from AH patients. Hierarchical clustering of the top 50% of differentially expressed genes (3789 genes) of GSE28619 show that gene expression in the liver of patients with AH clustered away from HC (Supplemental Figure 1; supplemental material available online with this article; https://doi.org/10.1172/jci.insight.141420DS1). A network heatmap was constructed from the pairwise correlation of differentially expressed genes and divided into color-coded modules (Supplemental Figure 1). The correlation between the module eigengenes (MEs) of HC and patients with AH was calculated to identify the modules that were highly related to AH (Supplemental Figure 2). The thickness of the clustering dendrogram branches represent groups of highly coexpressed genes, and the height of the branch is directly proportional to the number of genes in each cluster; the merged dynamic reduces the dimensionality of the modules by merging highly similar expression profiles (Supplemental Figure 2A). Module adjacency was also calculated to show the hierarchical clustering of the top 6 modules (Supplemental Figure 2B). From the results of the merged dynamic tree cut, the green (correlation, 0.95) and salmon (correlation, 0.92) modules were selected for further analysis, as their correlation to AH was greater than 0.9 (Figure 1A). Total membership of green and salmon modules is listed in Supplemental Table 1 and Supplemental Table 2. Module membership (MM) was correlated to gene significance for the green (Figure 1B) and salmon (Figure 1C) modules. A Venn diagram was then constructed to assess if members of the chemokine family of ligands and/or receptors were located in either module (Figure 1D). A total of 10 chemokine ligand and receptor genes was found to reside in the salmon and green modules. Within the salmon module, several chemokine ligand and receptor family members, including the entire *IL-8* family of chemokine ligands, *CCL2* and *CCL20*, were represented; in the green module was *CXCL10*, an ELR^–^ C-X-C chemokine (Figure 1E). Importantly, the differential expression for this subset of chemokines, therefore, was significantly associated with AH.

### Clustering analysis of RNA-seq data from patients with liver diseases of different etiology and severity identified a unique chemokine signature in severe AH.

**Figure 2:**
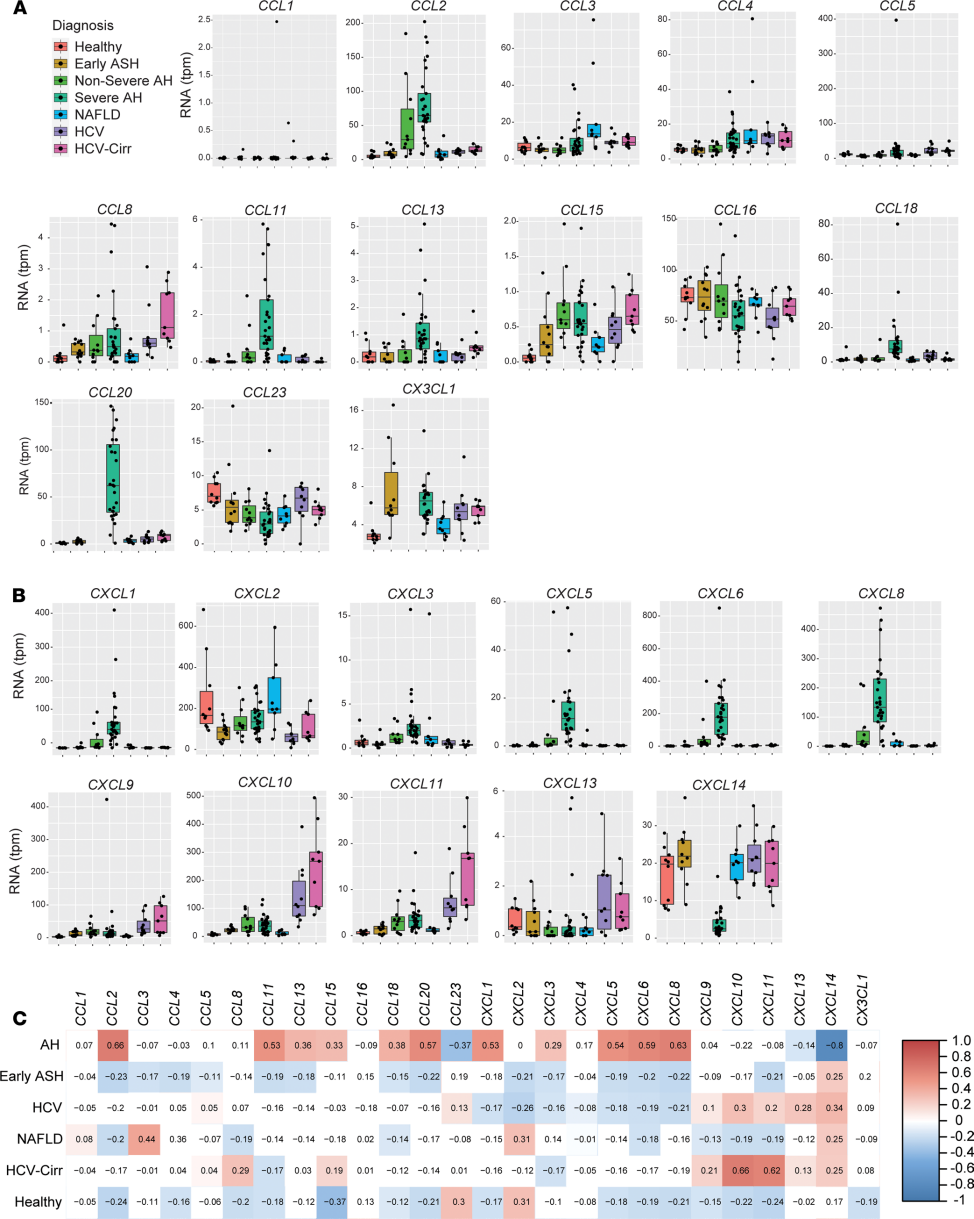
Chemokine expression and correlation with diagnosis from RNA-seq in livers of HC and patients with various hepatopathies. (**A** and **B**) Expression of C-C chemokines and *CX3CL1* (**A**) and C-X-C chemokines (**B**). Data are displayed as normalized transcripts per million (tpm) in box-and-whisker plots representing the mean, interquartile range (box), and upper and lower quartiles (whiskers) for HC (Healthy, *n* = 10), patients with early alcohol-associated steatohepatitis (Early ASH, *n* = 12), nonsevere alcohol–associated hepatitis (Non-Severe AH, *n* = 11), Severe AH (*n* = 29), nonalcoholic fatty liver disease (NAFLD, *n* = 9), hepatitis C virus (HCV, *n* = 9), or hepatitis C virus with cirrhosis (HCV-Cirr, *n* = 9). (**C**) Heatmap of Pearson’s correlation coefficients for expression of chemokine ligands to patient diagnosis with values represented in the boxes.

Since the data in GSE28619 show a direct comparison of healthy individuals versus patients with AH (4, 11), we next validated this coordinated expression of selected chemokines to AH in a second, distinct cohort of patients with AH and other chronic liver diseases (15). Published liver RNA-seq data were analyzed for the expression of chemokine ligands in the C-C (Figure 2A) and C-X-C (Figure 2B) families. As in the WGCNA of GSE28619 (Figure 1E), multiple chemokine ligands were also increased in patients with AH (Figure 2, A and B), including *CCL2*, *CCL11,* *CCL20*, *CXCL1*, *CXCL5*, *CXCL6*, *CXCL8*, and *CXCL10*. Correlation analysis demonstrated that changes in expression of multiple chemokine ligands were primarily associated with AH compared with other disease etiologies (Figure 2C).

The expression of some chemokine mRNAs in this second cohort also revealed that coordinated changes in expression were specific to AH, as compared with NAFLD and HCV. Expression of *CXCL10* was increased in patients with severe AH, as well as in patients with HCV either with or without cirrhosis (Figure 2B). In addition, expression of *CXCL2* mRNA was not changed in any patients, as compared with HC (Figure 2B). Interestingly, the strongest association with disease and hepatic chemokine mRNA expression was primarily found in patients with AH (Figure 2C), with Pearson’s coefficients above 0.5 for *CCL2*, *CCL11*, *CCL20*, *CXCL1*, *CXCL5*, *CXCL6*, and *CXCL8*.

**Figure 3:**
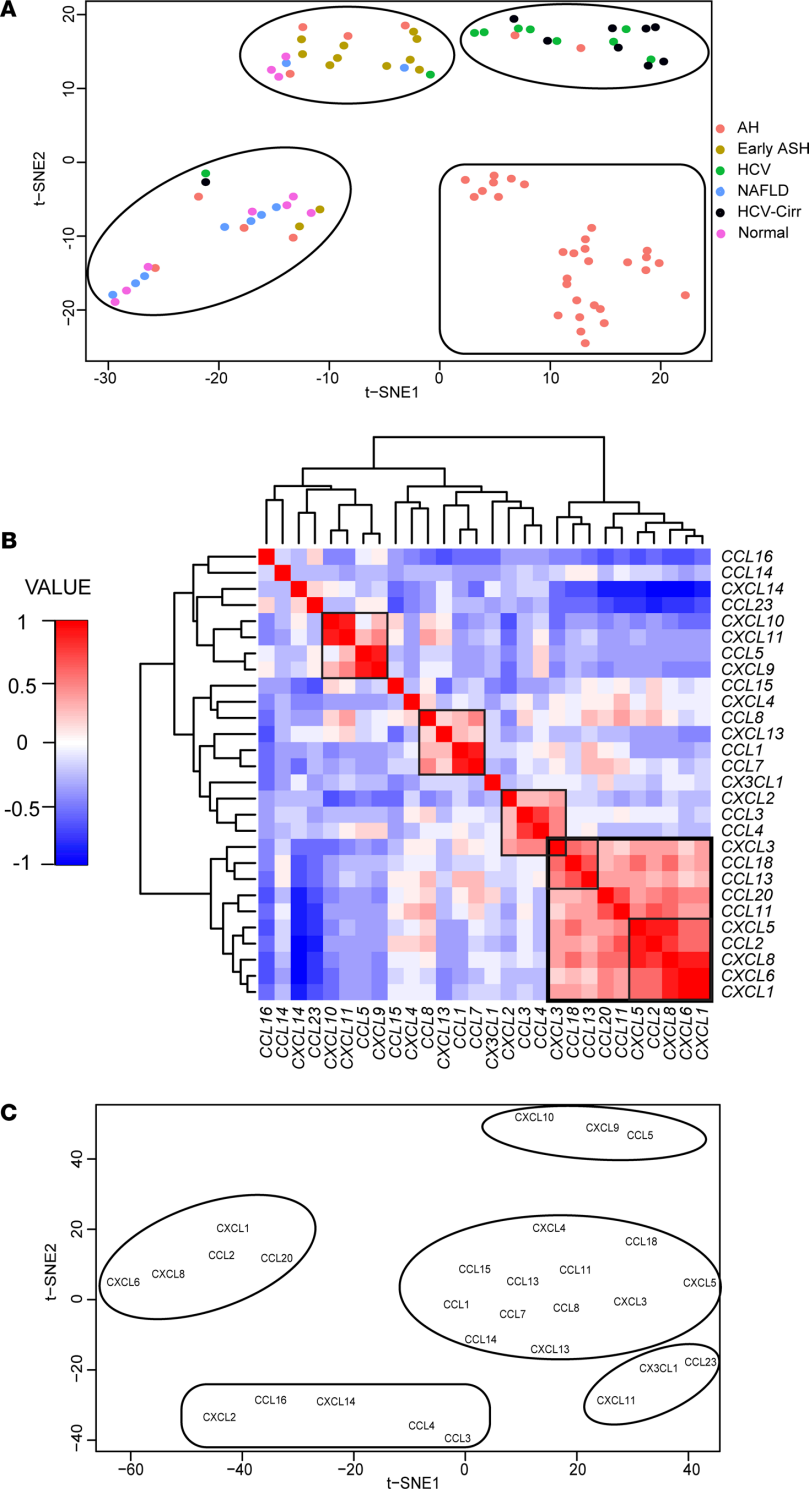
Clustering of chemokine expression data by t-SNE segregated patients with AH from other diagnoses and refined the hepatic chemokine signature in patients with AH. (**A**) Clustering of patients as determined by t-SNE of RNA expression for chemokines in livers of patients. (**B**) Heatmap of chemokine expression correlations in liver. (**C**) Clustering by t-SNE for expression of chemokine RNA in livers of patients from all hepatopathies.

Taken together, the analysis of chemokine ligand expression in these 2 separate data sets of gene expression in the livers of patients with AH suggests coregulation of specific chemokine ligands. In order to define this coordinated expression, clustering analysis of chemokine expression in the data set was performed by t-distributed stochastic neighborhood embedding (t-SNE). A cluster consisting of 30 of 40 patients with AH were segregated from all other diagnoses (Figure 3A). Most patients with NAFLD clustered with HC, and patients with HCV clustered either with or without cirrhosis (Figure 3A). When all AH patients were grouped together, the clustering suggested that regulation of hepatic chemokine expression has an AH-related etiology. Hence, all patients with AH were grouped together, irrespective of severity, for subsequent analyses.

Pairwise correlations for the expression of individual chemokine RNAs revealed that expression of several chemokines was significantly correlated in the liver (Figure 3B). Clustering of chemokine expression by t-SNE revealed that *CXCL1*, *CXCL6*, *CXCL8*, as well as *CCL2* and *CCL20*, were clustered apart from other chemokines (Figure 3C). Taken together, based on the analysis of these 2 cohorts, coordinated expression of specific chemokines consisting of *CXCL1*, *CXCL6*, *CXCL8* as well as *CCL2* and *CCL20* in liver was tightly coregulated in patients with AH.

### Hepatocyte-derived MIF directs hepatic chemokine expression after ethanol feeding.

**Figure 4:**
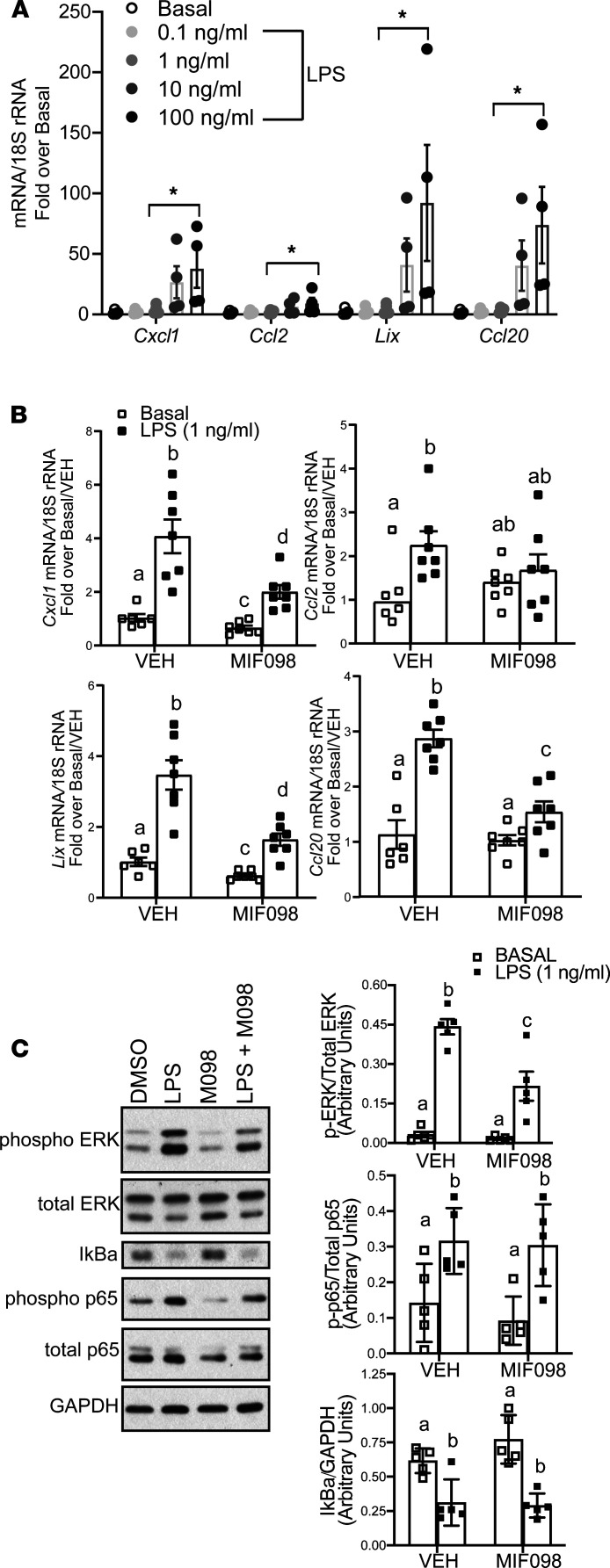
MIF is required for LPS-mediated upregulation of chemokine mRNA expression in response to LPS challenge. (**A**) AML-12 cells were treated with LPS at the indicated concentrations for 90 minutes, and expression of *Cxcl1*, *Lix*, *Ccl2*, and *Ccl20* mRNA was determined by qPCR. (**B**) AML-12 cells were pretreated with VEH (0.1% DMSO) or MIF098 (50 μM) prior to LPS challenge for 90 minutes. Expression of *Cxcl1*, *Lix*, *Ccl2*, and *Ccl20* mRNA was determined and normalized to Basal/VEH or LPS/VEH as indicated. (**C**) AML-12 cells were treated with 1 ng/mL LPS for 30 minutes, and phosphorylation of ERK and p65, as well as the abundance of IκBα, was determined by Western blot. GAPDH was used as a loading control. Values are expressed as means ± SEM. *P < 0.05 versus. BAS controls (*n* = 4-7). Means with different letters are significantly different, *P* < 0.05, by 2-way ANOVA with least square means multiple comparison tests. See complete unedited blots in the supplemental material.

The coordinated control of chemokine expression is not well understood, but MIF, a pleiotropic cytokine/chemokine, has been implicated in regulation of chemokine expression in a number of disease models, including ethanol feeding in mice (12–14). Importantly, MIF concentrations are increased in the circulation of patients with AH and are associated with patient morbidity and mortality, and accumulating evidence indicates that hepatocytes are an important source of MIF in patients with AH (13). We therefore utilized α mouse liver 12 (AML-12) hepatocytes to investigate the direct role of MIF in regulating expression of the ethanol-induced chemokine signature by hepatocytes. AML-12 hepatocytes readily release MIF into the culture media (13, 16); therefore, we made use of a small molecular MIF inhibitor, MIF098, to interrogate the impact of endogenously produced MIF on chemokine expression. When AML-12 cells were challenged with increasing concentrations of LPS for 90 minutes, the expression of chemokine mRNA was increased in an LPS dose–dependent manner (Supplemental Figure 3 and Figure 4A). The threshold for increased expression of *Cxcl1*, *Lix*, and *Ccl20* mRNA was at 1 ng/mL LPS, a concentration that is physiologically relevant in patients with severe AH (17, 18). LPS stimulation also rapidly increased the concentration of MIF in the media, detected as early as 30 minutes after challenge with LPS (Supplemental Figure 3). In order to interrogate the contribution of MIF to chemokine expression, AML-12 cells were treated with MIF098 (50 μM) during LPS challenge. When cells were treated with MIF098, basal expression of *Cxcl1* and *Lix* mRNA was decreased and LPS-mediated (1 ng/mL) expression of *Cxcl1*, *Lix*, *Ccl2*, and *Ccl20* mRNA was attenuated (Figure 4B).

We next investigated upstream signaling pathways that contributed to MIF-mediated increases in expression of chemokine mRNA. We targeted pathways common and downstream to both LPS and MIF after 30 minutes of LPS challenge. LPS increased phosphorylation of extracellular regulated kinase (p-ERK) I and II; this response was decreased by MIF098 coexposure (Figure 4C). LPS treatment also activated the NF-κB pathway, indicated by increased phosphorylation of the p65 subunit (p-p65) and decreased abundance of NF-κB inhibitor α (IκBα); however, this response was not impacted by MIF098 coexposure (Figure 4C). Pretreatment of hepatocytes with U0126 (10 μM), an inhibitor of ERK activation, also attenuated LPS-stimulated upregulation of chemokine expression in hepatocytes (Supplemental Figure 4), consistent with a role for this signaling pathway in the response to MIF. Taken together, these data demonstrate that MIF was required in coordinated chemokine expression in response to LPS in cultured AML-12 hepatocytes, consistent with the coordinated expression of chemokines observed in livers of patients with AH.

**Figure 5:**
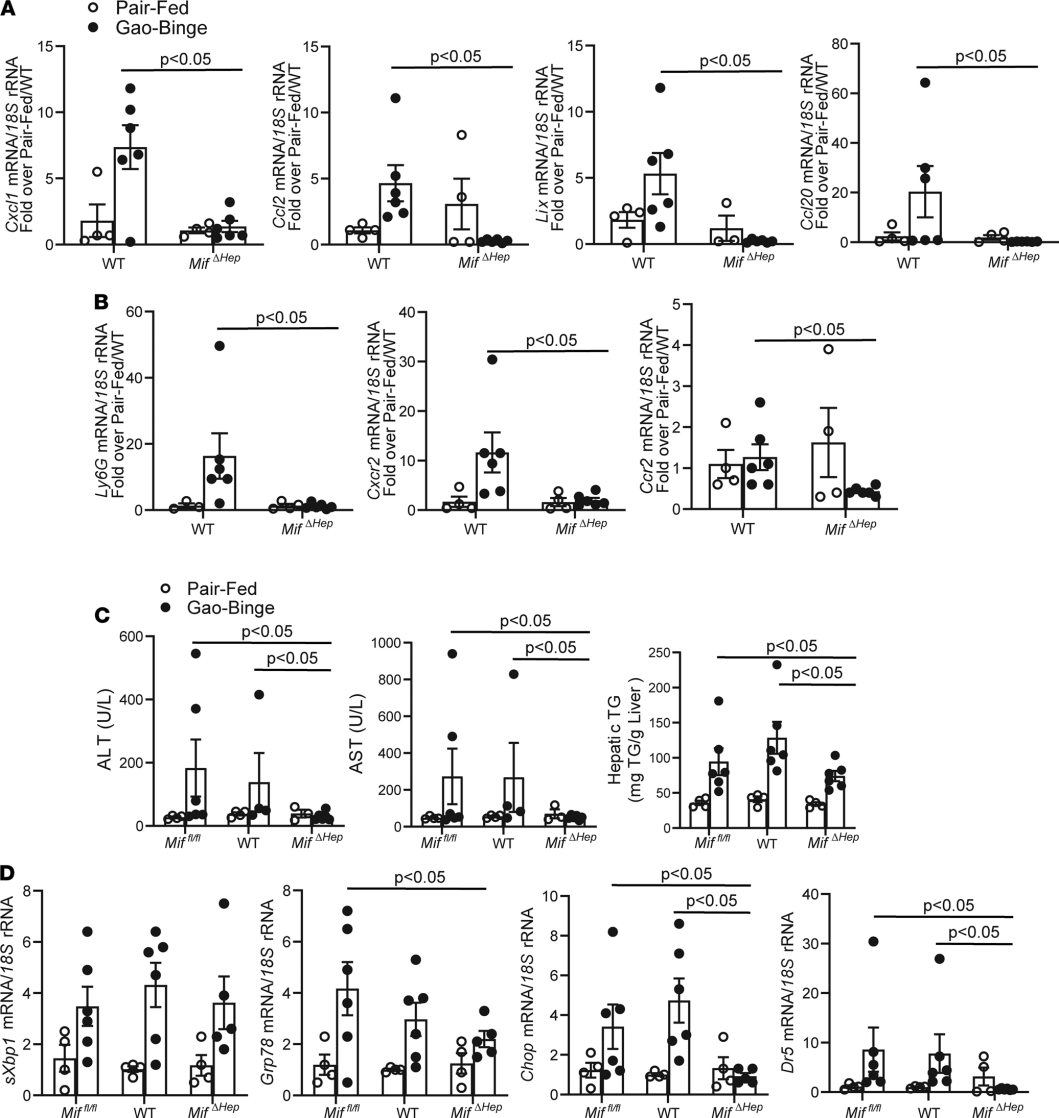
Hepatocyte-specific *Mif* deletion prevents Gao-binge–induced hepatocellular injury, steatosis, and expression of the chemokine signature. *Mif^fl/fl^*, C57BL/6, and *Mif^ΔHep^* mice were acclimated to a complete liquid diet and allowed free access to ethanol-containing (*n* = 5–6) or pair-fed (*n* = 4) control diets per Gao-binge feeding protocol. (**A** and **B**) Expression of *Cxcl1*, *Lix*, *Ccl2*, and *Ccl20* chemokine mRNA (**A**) and expression of neutrophil markers *Ly6G* and *Cxcr2* as well as monocyte surface marker *Ccr2* mRNA (**B**) was determined by qPCR in mouse livers. (**C**) ALT (U/L) and AST (U/L) activity in circulation was determined in plasma, and hepatic triglyceride content was measured in liver homogenate. (**D**) Expression of ER stress–associated mRNA for spliced *Xbp1* (*sXbp1*), *Grp78*, *Chop*, and *Dr5* was determined in mouse liver by qPCR. Values are expressed as means ± SEM. *P* < 0.05 versus pair-fed controls within genotype, by 2-way ANOVA with least square means multiple comparison tests.

We next tested if hepatocyte-derived MIF is important for the regulation of chemokine expression in livers of mice in response to Gao-binge ethanol feeding, a model of ethanol-induced liver injury associated with increased chemokine expression, exacerbated hepatocellular injury, and hepatic inflammation in mice (19). If hepatocyte MIF contributed to the coordinate regulation of chemokines in liver, then hepatocyte-specific *Mif* deficiency should prevent this response. Therefore, hepatocyte-specific KOs (Supplemental Figure 5) were generated (*Mif*^ΔHep^**) to test the hypothesis that hepatocyte-derived MIF drives coordinated chemokine expression after Gao-binge ethanol feeding in mice. Importantly, hepatic expression of the coordinated chemokine signature, *Cxcl1*, *Lix*, *Ccl2*, and *Ccl20* mRNA, was increased in WT mice but was completely prevented in *Mif*^ΔHep^** mice after Gao-binge feeding (Figure 5A). Lix is the murine homolog of human CXCL5 and CXCL6, and it is therefore a surrogate for both chemokines in mice (20, 21). A hallmark of Gao-binge ethanol feeding is increased neutrophil infiltration; Gao-binge–induced expression of mRNA for ELR^+^ C-X-C chemokines, important stimulators of neutrophil recruitment, was also completely prevented in *Mif*^ΔHep^** mice. Furthermore, expression of *Ccr2*, a receptor located on proinflammatory monocytes, was decreased in *Mif*^ΔHep^** mice, as compared with WT mice (Figure 5B). Therefore, hepatocyte-derived *Mif* was required for upregulation of the hepatic chemokine signature and leukocyte infiltration after Gao-binge ethanol feeding.

Importantly, *Mif*^ΔHep^** mice were protected from Gao-binge (Figure 5C), as well as chronic ethanol-induced increases (Supplemental Figure 6) in plasma alanine aminotransferase (ALT) and aspartate aminotransferase (AST), as compared with *Mif^fl/fl^* mice and WT mice (Figure 5C). Multiple arms of the ER stress pathways were induced in the liver by Gao-binge feeding in *Mif^fl/fl^* and WT mice, including increased splicing of x-box protein 1 (*sXbp*1) mRNA and increased expression of glucose-regulated peptide 78 (*Grp78*), C/EBP homologous protein (*Chop*), and Chop-dependent gene death receptor 5 (*Dr5*) mRNA. Gao-binge also induced *sXbp1* and expression of *Grp78* mRNA in *Mif*^ΔHep^** mice, but it did not induce expression of *Chop* and *Dr5* mRNA (Figure 5D). Hepatocyte-derived *Mif* expression was required for ethanol-induced liver injury, steatosis, and cellular stress, directly connecting hepatocyte-derived MIF as necessary to drive ethanol-induced liver injury and inflammation.

## Discussion

Inflammation is associated with the onset and progression of almost all chronic diseases, including ALD and other liver diseases (6, 22). The current study highlights critical changes in expression of chemokines in the livers of patients with AH and how changes in expression of multiple chemokines might be controlled in patients with severe AH. Canonical functions of chemokines and MIF in ALD are associated with leukocyte infiltration into the liver, including monocytes, macrophages, and neutrophils (23–25). There is some controversy to what roles leukocytes play in the progression of ALD in humans; the accumulation of neutrophils in the livers of patients with AH has been associated with both favorable and negative outcomes for patient morbidity and mortality (25–28). Since current therapeutic options for ALD are limited and ineffective in nearly half of patients, a complete understanding of the underlying inflammation will optimize mechanistic discoveries in the future. In the current study, we investigated how the dynamics of the chemokine ligand family changed transcriptionally throughout ALD progression, and we discovered a coordinated upregulation of *CXCL1*, *CXCL5*, *CXCL6*, *CXCL8*, *CCL2*, and *CCL20* expression that was tightly correlated to patients with AH and was controlled by hepatocyte-derived MIF in ethanol-fed mice. Understanding how this signature is upregulated in ALD has the potential to provide meaningful therapeutic insight into the specific pathophysiology of ALD compared with other liver diseases.

The WGCNA method of analysis identified modules of differentially expressed genes that had the strongest correlation to AH as compared with HC. The analysis was unsigned; therefore, the opposite signs of the correlation values — 0.95 and –0.92 for green and salmon, respectively — do not provide insight regarding whether members of the modules would contribute to or protect from AH pathogenesis. However, the opposite signs do suggest that overlap between module members was unlikely (Figure 1A, Supplemental Table 1, and Supplemental Table 2). The chemokines identified are upregulated in patients with AH as compared with HC when measured individually, consistent with other studies (3, 4).

We next extended the analysis into a different cohort, which included patients who drink alcohol to excess with early, nonsevere ALD, through those with severe AH who required liver transplant (Figure 2 and Figure 3). While the 2 analyses were generally consistent with each other, there were some modest differences that could be attributed to the sensitivity of the technique (microarray versus RNA-seq), the quality of the sequencing, or variations stemming from sample collection or storage (29). Another finding from this second cohort suggests that hepatitis as a diagnosis, irrespective of etiology, is not sufficient to drive enhanced chemokine expression. Consistent with other reports, patients with HCV had far fewer changes in expression of chemokines except for *CXCL10* and *CXCL11* (Figure 3) (1, 2, 30). In patients with NAFLD, no significant changes in expression of chemokines were detected as compared with HC. Interestingly, in autoimmune hepatitis, a chronic liver disease with a known connection to MIF, hepatic production of chemokines are primarily T cell chemotactic factors outside of the IL-8 family (31, 32). Overall, patients with AH exhibited the most changes, both in number and magnitude, in expression of chemokines compared with other liver diseases.

While many chemokines were upregulated in patients with AH, clustering analysis revealed a distinct and specific signature of *CXCL1*, *CXCL6*, *CXCL8*, *CCL2*, and *CCL20*, suggesting that these chemokines may be coregulated. The chemokines in this group (Figure 3) are largely localized to the same genomic neighborhood (within 4 Mb) for either the C-C (Chromosome 17) or C-X-C (Chromosome 4) chemokine families (33). In contrast, *CCL20* (Chromosome 2) is a standalone gene with regard to its genetic locus within the chemokine family. These data suggest that upregulation of this chemokine signature was not likely due solely to the proximity of chemokine genes to one another. An interesting finding was the negative regulation of *CXCL14* specifically in AH; CXCL14 is an ELR^–^ C-X-C chemokine similar to CXCL10 (Figure 3C). Clustering analysis, however, did not include *CXCL14* with the other chemokines, so we did not continue to include this particular chemokine for the remainder of the study. Taken together, the analysis from the chemokine expression in these data sets identified an hepatic chemokine signature in patients with AH that was distinct from patterns of chemokine expression in other hepatopathies, suggesting that there was a common upstream mechanism directing this phenomenon.

In considering a potential mechanism for expression of this chemokine signature in AH, we hypothesized a specific role of the pleiotropic cytokine-chemokine MIF in regulation of chemokine expression. In patients with AH, expression of MIF protein is upregulated in hepatocytes. The concentration of MIF is elevated in suprahepatic sera and is associated with higher mortality in patients with AH (13). The marked upregulation of MIF protein in livers of patients with severe AH is not paralleled with increased expression of *MIF* mRNA (14). This has been demonstrated in several studies with alcohol feeding in mice and in human tissues (12–14). Furthermore, within the current study, expression of *MIF* mRNA was not increased in patients with AH in GSE28619 or in the second cohort of patients with alcohol-associated steatohepatitis (ASH) and other hepatopathies (data not shown). Although *MIF* mRNA is modestly upregulated in many diseases, including AH, the large, preformed pools of MIF protein that are stored in cells and ready to be released in response to inflammatory or noxious stimuli are likely of greater significance to the pathophysiology of the disease (34, 35). Release of MIF is likely a danger signal in AH, especially when AH is severe.

Interestingly, the current study identified that the greatest increase in magnitude for expression of chemokines and the coordinated regulation of expression was found in patients with severe AH (Figure 3C), and this is paralleled by hepatic MIF protein expression and release in patients with AH (13, 14). MIF is a known regulator of chemokine expression following ethanol feeding with or without binge or chemically induced liver fibrosis by carbon tetrachloride, and in other cell types including endothelial cells, macrophages, and hepatocytes (Supplemental Figure 6) (13, 16, 36, 37). What is even more compelling about hepatocyte-derived MIF controlling chemokine expression is that hepatocytes are a pivotal source of many chemokines, like CCL2 and the IL-8 family, in liver diseases, including AH (2, 16, 38). If MIF is released by hepatocytes in patients with severe AH, then it could represent an autocrine/paracrine feedback loop leading to exacerbated liver inflammation and mortality in patients with AH. Furthermore, the current study strongly suggests that MIF is a contributor to inflammation and injury in AH compared with NAFLD and HCV. This disease-specific role of MIF in patients with AH is consistent with data from murine models, where MIF contributes to ethanol-induced liver injury (12–14) but may protect from high-fat diet–induced liver injury and chemically induced liver fibrosis (39, 40). Additional evidence for context-specific roles of MIF was reported in a recent study, wherein the use of hepatocyte-specific *Mif* KOs revealed a previously unknown profibrotic effect of MIF in a diet-induced model of fibrosis in mice, contrasting with previous results using global *Mif* KOs (41). Taken together, the complex biology of MIF in liver diseases is multifaceted and context dependent.

While chemokine expression is typically associated with inflammatory cells, they are also abundantly expressed in epithelial cells, including hepatocytes (1, 16). The findings after Gao-binge ethanol feeding in the *Mif*^ΔHep^** mice added strong evidence that hepatocyte-derived MIF was sufficient to drive ethanol-mediated hepatocellular injury and liver inflammation. *Mif* deficiency only in hepatocytes was sufficient to prevent increased hepatocellular injury, cytotoxic arms of ER stress, immune cell recruitment, and most importantly, expression of the chemokine signature after Gao-binge ethanol feeding (Figure 5). The results from *Mif*^ΔHep^** mice following Gao-binge feeding and cultured hepatocytes presented here add to the growing body of evidence that hepatocytes are a critical source of MIF in ALD (12, 13).

It is worthy of note that the magnitude of expression for hepatic chemokine mRNA after Gao-binge in mice is much lower than that observed in patients with AH. This may be related to species differences and/or differences in disease severity. In addition, murine and human chemokines differ; for example, mice do not express *CXCL8*, and the closest murine form of *CXCL5* and *CXCL6* is *Lix*. Furthermore, although Gao-binge ethanol feeding is a useful model of ethanol feeding that induces neutrophil accumulation in the hepatic parenchyma, the magnitude of accumulation is markedly lower than in patients with AH (13, 14, 42). Despite these differences, the same coordinated pattern of expression for hepatic chemokines is paralleled both in murine models and in patients with AH.

The current study demonstrates why targeting a single chemokine or receptor in AH would be problematic and ineffective, as redundancy and overlap in action are important features of the chemokine system. The difficulty in identifying effective therapeutic targets in ALD is likely related to the multiplicity of factors acting concurrently in the pathophysiology of ALD. Furthermore, given the cell-autonomous roles of factors such as MIF or chemokines, it is likely that therapeutic targeting to both the source and site of action will be required for refined interventions. Therapeutic targeting of MIF or MIF-dependent signaling in hepatocytes might lead to a better outcome in patients with severe AH.

## Methods

### WGCNA of differentially expressed genes in GSE28619.

Gene expression analysis in the liver of 7 HC and 15 patients with AH was used in the unsigned WGCNA with publicly available gene expression database GSE28619 (https://www.ncbi.nlm.nih.gov/geo/query/acc.cgi?acc=gse28619). Patient characteristics and study approval were detailed in a previous publication (4). The top 50% of differentially expressed genes (3789 genes) were selected to construct an unsigned, coexpression network using the WGCNA package in R, version 3.6 (43). The power of β = 10 (scale-free *R^2^* = 0.8) was selected as the soft thresholding parameter to ensure a scale-free network. The minimum module size was set to 30, and the threshold for merging similar modules was set to 0.25. The correlation between MEs and clinical traits was calculated to identify the modules that were highly related to AH. Modules with absolute values of MEs above 0.9 were selected. Genes with an absolute gene significance (GS) value above 0.5 and an absolute MM values above 0.6 in green (852 genes) and salmon (1852 genes) modules were regarded as hub genes for further analysis (Supplemental Table 1 and Supplemental Table 2).

### RNA-seq database and clustering analysis.

RNA-seq data from livers of patients with various hepatopathies were acquired in-house; patient characteristics from this cohort are found in ref. 15. In brief, the patients in this cohort are either HC, heavy drinking patients with diagnoses ranging in severity from early alcohol-associated steatohepatitis (early ASH), through patients with AH receiving liver transplant, patients with NAFLD, and patients with HCV without or with compensated cirrhosis (HCV or HCV-Cirr). Chemokine expression was supplied as normalized transcripts per million (tpm). Clustering analysis of RNA expression was performed via t-SNE using the Rtsne function in R, version 3.6. Pearson’s correlations and heatmaps were generated from normalized RNA expression data and compared with patient diagnosis or as pairwise correlations in R.

### Generation of Mif^ΔHep^ mice.

A breeding colony of *Mif^fl/fl^* mice (44) on a C57BL/6 background was established and maintained at the Cleveland Clinic. ALB-CRE–expressing mice were purchased from The Jackson Laboratory and crossed with *Mif^fl/fl^* mice to generate *Mif*^ΔHep^** mice. The genetic background of the mice was determined by genotyping 148 distinct SNPs by the Jackson Laboratory’s SNP genome scanning analysis. *Mif^fl/fl^* mice were on a mixed C57BL/6J and C57BL/6N background in an approximate 45:55 ratio of C57BL/6N:C57BL/6J, whereas the *Mif^Hep^* mice were a 30:70 ratio of C57BL6/N:C57BL/6J (Supplemental Table 3). To properly control for these differences in genetic background, C57BL/6N mice were purchased from Charles River Laboratories and crossed with C57BL/6J for 1 generation, generating mice with a 45:55 ratio of C57BL/6N:C57BL/6J mice. The F1 generation was backcrossed once more to C57BL/6J mice to generate a strain with a 25:75 ratio of C57BL6/N:C57BL/6J (abbreviated as WT) (Supplemental Table 4).

### Gao-binge feeding model.

Gao-binge ethanol feeding was carried out as previously described, with minor modifications (19, 42). On day 11, pair-fed and ethanol-fed mice (5% ethanol v/v) were gavaged with an equivalent volume of 5 g/kg maltose or 5 g/kg ethanol (Pharmco, Greenfield Global USA Inc.) in water, respectively. Mice were anesthetized at 6 hours after gavage, blood was collected in nonheparinized syringes from the posterior vena cava, livers were excised after a brief perfusion, and mice were euthanized by exsanguination. Some mice were also fed 5% ethanol (v/v) for 10 days but not subjected to the ethanol binge. Portions of each liver were fixed in formalin or frozen in OCT compound (Sakura Finetek) for histology, flash frozen in liquid nitrogen to be stored at –80°C for analysis at a later time. Blood was transferred to EDTA-containing tubes for plasma isolation; plasma was isolated and stored at –80°C until further analysis.

### RNA isolation and quantitative PCR (qPCR).

Flash-frozen liver was homogenized in Qiazol (Qiagen), and RNA was isolated using the Direct-zol RNA Kit (Zymo Research). Liver RNA was reverse transcribed by SuperScript VILO cDNA Synthesis Kit (Thermo Fisher Scientific). The relative mRNA was determined using primers listed in Supplemental Table 5 by the ΔΔCt method, normalized to 18S rRNA, on a QuantStudio5 qPCR machine.

### AML-12 cell culture.

The murine hepatocyte cell line, AML-12, was purchased through the American Tissue Culture Collection (ATCC) and grown in DMEM:F12 Medium supplemented with 10% FBS, 10 μg/mL insulin, 5.5 μg/mL transferrin, 5 ng/mL selenium (Invitrogen), and 40 ng/mL dexamethasone (MilliporeSigma) (complete medium). For experiments, cells were acclimated to complete medium without dexamethasone for 18 hours prior to stimulation with bacterial LPS (Thermo Fisher Scientific). AML-12 cells were treated with 50 μM MIF098 (45), ERK activation inhibitor U0126 (Cell Signaling Technologies), or vehicle control (0.1% DMSO; VWR Chemicals LLC) 1 hour prior to LPS challenge at the indicated concentrations. RNA was isolated with the Direct-zol RNA Kit (Zymo Research), and protein was isolated as previously described (14). Cell lysates were separated on 10% polyacrylamide gels and used for Western blot analysis with antibodies against phospho-ERK1/2 (sc-7383, Santa Cruz Biotechnology Inc.), phospho-p65 (3033S, Cell Signaling Technology), total ERK (06-182, MilliporeSigma), total p65 (6956S, Cell Signaling Technology), and IκBα (9242, Cell Signaling Technology). GAPDH (MAB374, MilliporeSigma) was used as a loading control. Signal intensities were quantified using ImageJ (NIH).

### Biochemical assays.

Plasma ALT and AST activities were assayed with enzymatic assay kits from Sekisui Diagnostics, per manufacturer instructions. Liver triglycerides were determined by assay kits purchased from Pointe Scientific Inc.

### Statistics.

Two-way ANOVA was performed using the general linear models procedure (SAS). Data were log-transformed if necessary to obtain a normal distribution. Post hoc comparisons were made by least square means testing. *P* values of less than 0.05 were considered significant.

### Study approval.

Animal protocols were approved and conducted in accordance with the Cleveland Clinic IACUC regulations (approval no. 2017-1885). For human samples, written and informed consent was obtained for all patients as noted in previous publications (11, 15). Dataset GSE28619 was obtained from the gene expression omnibus (ncbi.nlm.nih.gov/geo). The RNA-seq data set is available from the Database of Genotypes and Phenotypes (dbGAP) of the National Center for Biotechnology Information under accession no. phs001807.v1.p1 (15).

## Author contributions

KLP, XF, MRM, and LEN conceived, designed, and directed the studies with input from LL, R. Bucala, MVC, JA, and R. Bataller. KLP wrote the manuscript with assistance from all other authors. KLP and XF performed the WGCNA on GSE286129 and analysis of phs001807.v1.p1. KLP, CDK, EH, XW, and MRM performed the mouse studies, in vitro cell work, qPCR, and Western blot analysis.

## Supplementary Material

Supplemental data

Supplemental Table 1

Supplemental Table 2

Supplemental Table 3

Supplemental Table 4

Supplemental Table 5

## Version 1 05/04/2021

In-Press Preview

## Version 2 06/08/2021

Electronic publication
