# Supplementary material for: Role of MIF in coordinated expression of hepatic chemokines in patients with alcohol-associated hepatitis
Source: JCI Insight. 2021 Jun 8;6(11):e141420. doi: 10.1172/jci.insight.141420 (PMC8262327; doi:10.1172/jci.insight.141420)
Supplement: Supplemental data [file jciinsight-6-141420-s151.pdf]

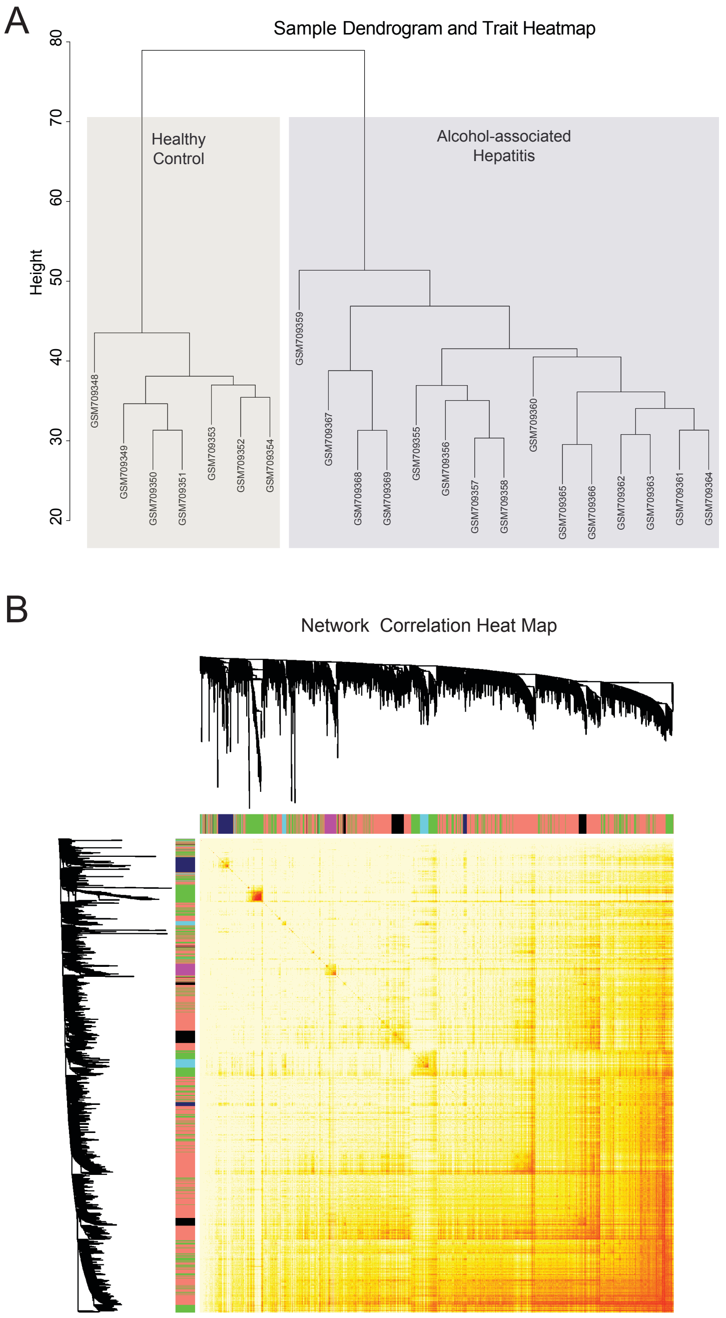

Supplemental Figure 1. Construction of the WGCNA Network in GSE28619. A) Hierarchical clustering of Healthy Control (HC) (n=7) and patients with Alcohol-associated Hepatitis (AH) (n= 15) in GSE28619 as determined by the top 50% of differentially expressed genes. B) Network correlation heatmap and establishment of color-coded modules of gene members.

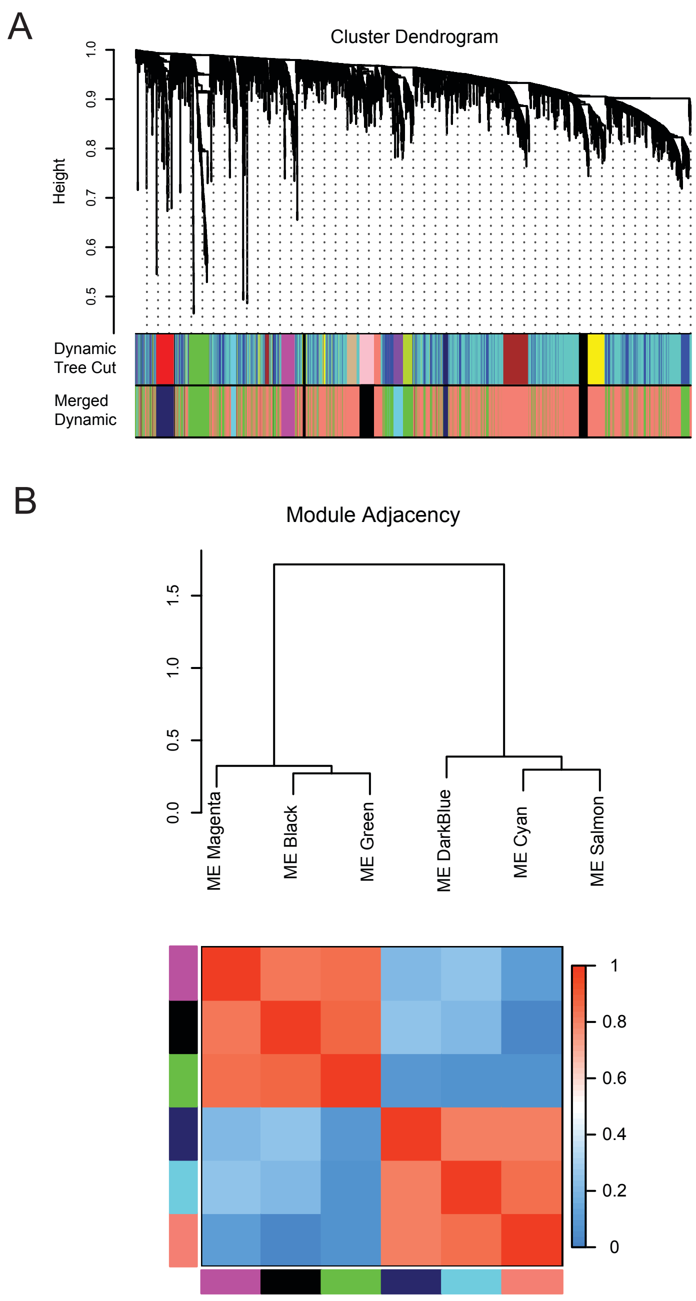

Supplemental Figure 2. Gene clustering and module formation by WGCNA. A) Cluster dendrogram for colored gene modules generated by WGCNA identified the highly coexpressed genes in each branch. B) Module adjacency heatmap in WGCNA detailed how related each module is to each other.

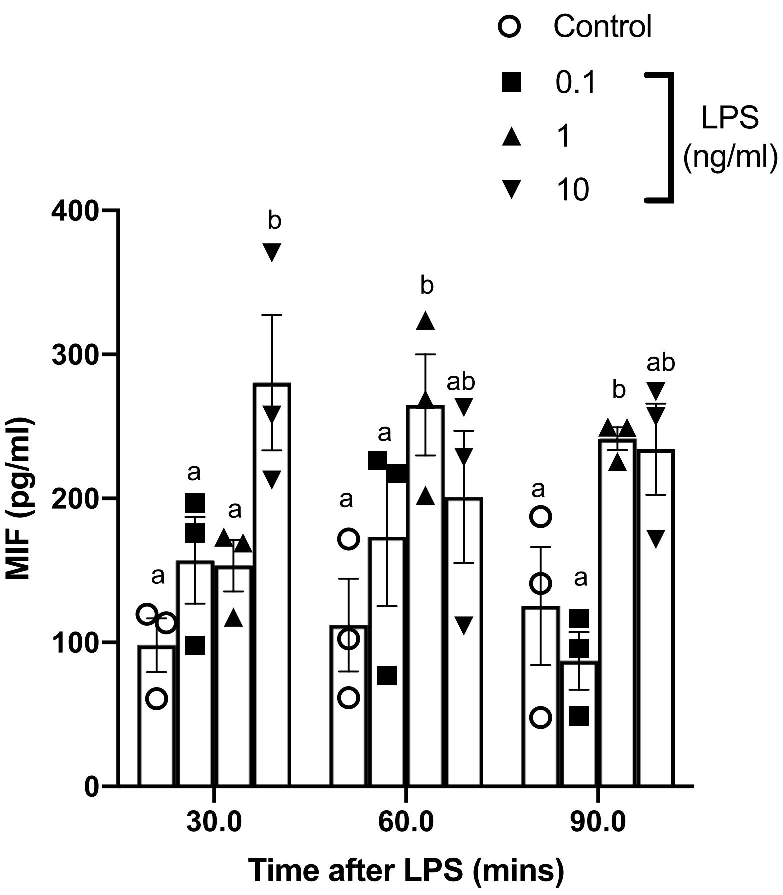

Supplemental Figure 3. Release of MIF protein from AML-12 Cells after LPS Challenge. AML-12 cells were challenged with increasing concentrations of LPS for the times indicated and culture supernatants were analyzed by ELISA for relased MIF. Data are represented as means ± SEM. Means with different letters are significantly different, $p<0.05$.

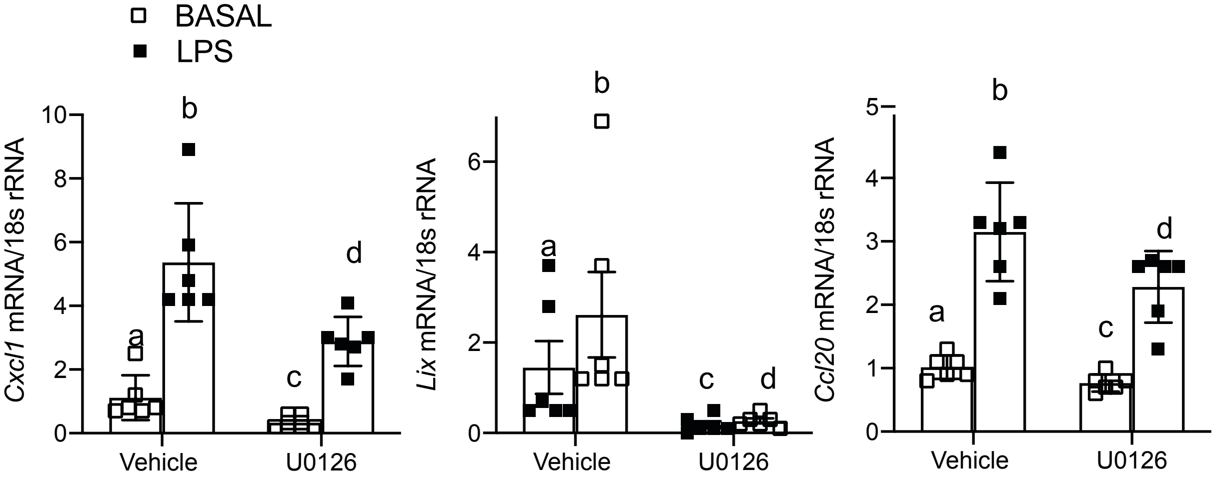

Supplemental Figure 4. Role of ERK signaling in LPS-mediated chemokine expression in AML-12 cells. Cells were pretreated with ERK activation inhibitor U0126 (10 μM) for 60 minutes prior to LPS challenge (1 ng/ml) for 30 minutes. Expression of mRNA for *Cxcl1*, *Lix* and *Ccl20* were determined by qRT-PCR. Data are represented as means ± SEM. Means with different letters are significantly different, $p<0.05$.

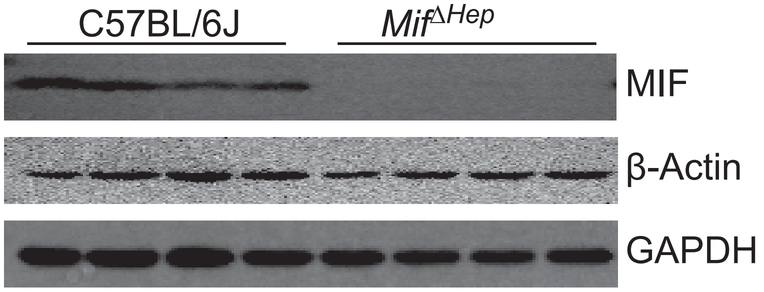

Supplemental Figure 5. Hepatocyte-specific Mif deletion significantly reduces MIF protein expression in the liver. Livers from C57BL/6J or Mif$^{\Delta Hep}$ mice were homogenized in lysis buffer and separated on a 12% SDS-PAGE gels. Blots were probes for MIF, b-Actin and GAPDH.

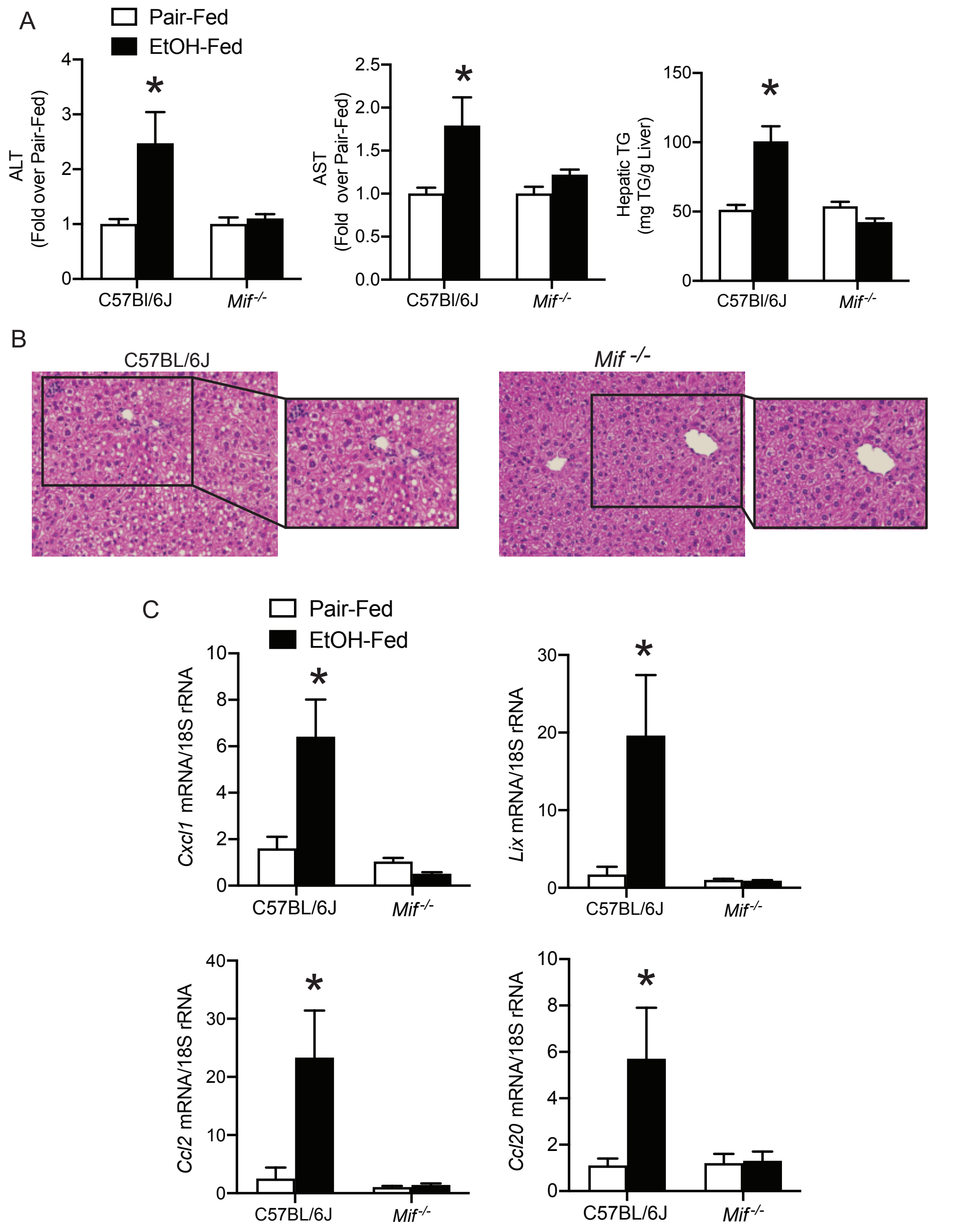

Supplemental Figure 6. Liver Injury, Steatosis and Chemokine Expression are MIF-dependent after Chronic Ethanol Feeding. C57BL/6J or *Mif*$^{-/-}$ mice were allowed free access to a liquid diet containing 5% v/v (28% total kCal) ethanol or control diet for 10 days. A) Plasma ALT (U/L) and AST (U/L) and hepatic triglyceride were determined by biochemical assay. B) Formalin-fixed, paraffin-embedded liver sections were stained by hematoxylin and eosin. C) Livers were homogenized in Trizol and liver mRNA expression for Cxcl1, Lix, Ccl2 and Ccl20 were determined by qRT-PCR. * - $p<0.05$ vs. pair-fed controls.
