# Supplementary material for: Role of MIF in coordinated expression of hepatic chemokines in patients with alcohol-associated hepatitis
Source: JCI Insight. 2021 Jun 8;6(11):e141420. doi: 10.1172/jci.insight.141420 (PMC8262327; doi:10.1172/jci.insight.141420)
Supplement: Supplemental Table 1 [file jciinsight-6-141420-s152.pdf]

GXYLT1
NPLOC4
LOC101927204
GLTP
MPP5
TPR
RAB11FIP5
GLS
CIB1
ROGDI
FAM13A-AS1
MSRA
SCAND1
MRPL51
ZCCHC14
ZNF521
THBS3
DYSF
CD2BP2
KPNB1
ABCC5
ATL2
CNKSR3
SATB1
TRPT1
SDAD1
ZBED1
UTP3
DHPS
RNPEP
ZHX3
LIMS2
TUBA4B
ITGAL
MYCT1
KARS
REPS2
C19orf60
RPS5
ZYG11B
AK6
HIBADH
LAMTOR5

CNN3
SMDT1
CFHR2
RPS12
ABCB10
FAM99B
SCPEP1
FAM107B
ZNHIT1
PPM1A
DCAF6
ELP2
BNIP3L
CXorf23
USF2
ZNF510
CMC4
C15orf61
MLKL
PRKAG2-AS1
ITGA1
SEC62
RAB22A
CCDC86
COPG2
HDAC1
EIF5A2
SPTBN2
IFT52
VPS72
GPR155
DPYD
SLC17A9
DMD
C17orf58
PHPT1
PIP4K2C
RTTN
SLBP
MTFR1
FGF14-AS2
MYO1C
COMMD2

ASRGL1
HERPUD1
GLG1
LEPRE1
SEC23B
TRAK2
IL1R1
TMEM11
ZNF260
TRAPPC6A
GLCCI1
LHX2
C12orf66
CMYA5
MOB1B
LRRFIP2
FAM210A
SDR39U1
ITPA
ACTN1
ZNF330
MPV17L
ATP6V1C1
SH3GLB1
TAX1BP1
ENC1
SCRN2
CFI
GORASP2
BDH1
DDX20
HPX
PPFIBP1
CCNJ
NUCKS1
CP
ARPC5L
LYRM5
RANBP9
ZFP36L1
NUBP2
ECI2
PEBP1

SNRPG
LINC01420
ZDHHC11
MRPL3
SLC45A3
8-Sep
CREBRF
NHP2
ZMAT1
CHD4
RAP1GAP
AGFG1
B4GALT1
SMEK1
RPF2
RPL29
PRPF38A
RP11-792A8.4
CHSY3
PLEKHB2
NAPEPLD
EIF3A
PHF5A
FBXO31
NDUFA12
GATSL2
HEXIM2
CROCCP2
VCL
CNOT11
MASP2
TP53I13
CREBZF
PPP1R35
IFT46
ASGR1
FAM184A
EIF3I
YTHDF2
ABCC1
ZBTB20
PLA2G16
RP11-410L14.2

KCTD3
CDK5RAP2
RNF128
CCDC104
IGLJ3
FBXO21
USP38
ELFN1
TRMT1L
ETF1
LOC102725343
TFDP2
SMARCD2
PPTC7
CLK1
ZFYVE26
ABCC10
KLHDC10
ATG9A
LYRM7
GNG5
ARPP19
ARHGAP35
NALCN
ZFP1
NMRK1
PANK3
PPP1R1C
RARS
PITPNA
SIVA1
USP4
WDFY1
ABCA8
IFT80
SMARCAL1
MOB1A
ODF2
BLVRA
LSM1
CALU
VEGFB
2-Mar

TFPT
TPRG1
MET
MRI1
PABPC3
ZNF546
USPL1
SMS
LOC101930405
RP4-595K12.1
CTAGE5
RAB11A
RPL10A
SCAPER
VDAC2
FAHD2A
LRRC2
LPPR1
GNB1
C19orf10
OPN3
PDRG1
PSMG2
ABCA11P
CIPC
MED30
IFNAR2
NT5C3B
DCN
DEPDC5
ERI1
KIAA1279
FAM57A
COBL
RPS6KA3
AVEN
TSG101
SCML2
STAM
F12
DIP2C
TRIP4
SSFA2

MMD
NINJ1
GNS
ESPN
PER3
KLHDC2
ANKRD28
C14orf166
TOM1L1
SRP14
RPL12
NBR2
TBL1X
MBLAC2
CCDC150
DOK6
TTL
EDF1
LAMTOR1
CYFIP1
SLC46A1
ARHGAP12
PGRMC1
FJX1
RABIF
LIN7C
SLC9A6
SLC5A3
GTF2E2
MFSD12
TMEM41B
PAQR4
DCTPP1
NAB1
CREB3L4
SNTB1
CES2
MTSS1
MPZL1
ZNF436
TRIM27
SIK3
ZDHHC13

SRSF5
RP11-258C19.7
ENAH
RSBN1L
CIAPIN1
PLA2G12A
CTNNBL1
AGMAT
MOB2
UBE2E1
TRIM24
POLR3C
MAP2K6
NOL4
TGFBR1
DIEXF
FLII
SH3RF3
KLHDC3
ATPAF1
S100A16
C1orf109
EIF6
MSN
KLHL42
KIAA0196
ADCK3
SH3BP2
STK17A
KPNA4
NPTN
MMAB
OSBPL1A
CRAT
RAPGEF2
FBXL16
RP3-507I15.1
C8orf46
ALKBH6
SOCS6
NDUFA5
FMR1
YWHAG

NGFRAP1
SLC25A27
SOX5
SSR2
DCTN6
PDE11A
GPD1
PLA2G4C
UBE2A
SKIV2L
METTL1
CDIPT
PTMS
NAT1
DNM1L
SWAP70
OSTM1
CLYBL
TMEM65
ZBTB18
AC083843.1
CYP4V2
RIPK2
GPN1
HSDL2
LETMD1
LINC00476
ECI1
F9
NRP1
FKBP14
SLC20A2
GEN1
MICU2
TJP3
TMEM133
POMP
EPN1
ABHD12
DYRK4
BCKDK
PRELID1
XYLB

BCKDHA
LINC00526
PRR5
LITAF
SLC10A3
DCBLD1
CIRH1A
ACVR2B
GPRIN3
NIFK
MST1
KAL1
MCEE
MAN1A1
RAI14
PDCD10
SORBS3
PPP1R3E
UBE2N
THAP7
RHOC
SMARCA2
LARP1B
CCNDBP1
SLC2A2
LACTB
SLCO2B1
DHRS7
METTL7A
C8orf76
ACY3
A1BG
GGACT
LOC100310756
LEPREL4
AGTR1
POGLUT1
CCT5
MBTD1
RCC1
WDYHV1
RGAG4
TRMT6

SENP2
KIAA1958
FEZ1
ASAH1
SIRT5
AIM1
PDE4DIP
SPON1
APOH
LOXL2
BEND3
IGKC
PTRF
C9orf16
NRAS
LOC101927752
HLA-A
L2HGDH
IMPDH2
WDR81
AMPD2
SLC25A22
RSAD1
KLF12
CLCN5
GOLPH3
RP11-339B21.15
TXNDC9
PCYOX1
WDR54
MPLKIP
CDK4
NAT10
EPS8
CUTC
NAA60
SFXN3
PTPRD
MYO5A
SMIM19
KLF3
PSMG3
DBN1

ATP6V0D1
NARS2
AMOTL1
PNRC2
KDM8
PIGV
PACSIN2
ATP2B4
RALB
MITF
FAM129B
HYAL2
PRDX6
PRNP
RDH5
KLHL29
THAP10
SNRPD1
ABHD10
PAFAH1B3
PGAM1
ART4
ST13
EXOC1
FAM13A
ECSIT
MPST
TCF4
XRCC5
MPC2
AGBL2
GNG12
TBC1D17
GET4
PTPRM
USP31
PHLPP1
SFT2D2
ACAA2
YBX3
MTFR1L
GRTP1
HEPH

GPR180
NR3C1
LINC01314
DTNA
MPP1
ABCA5
YWHAB
ACBD3
NIPSNAP1
SARDH
MED10
SIMC1
NDUFAF4
CEP120
KIF5B
POLE
CDK7
MTHFD2L
SLC31A1
SFXN1
SH3RF2
CTNNA1
SFXN2
APH1B
SLC41A3
HNF4A
PPCDC
SUB1
NPM1
PLS1
MRPS21
MED9
BC022047
NOB1
TMEM64
GALNT18
RRAS2
ANKRD46
JAKMIP2
RBX1
DAXX
C12orf49
SUMF2

CLEC5A
ADPRHL2
C8A
IQSEC1
SEPP1
TMEM251
DNMT1
PRKRA
C14orf28
ACADM
DHRS4-AS1
C1S
SLC37A3
LGALS8
SIRPA
IFITM2
ZFAS1
C14orf119
POLR3GL
ESF1
EFTUD1
CEBPZOS
TRIP6
CDC14B
F2
IDNK
TCEA3
AHCY
CMC2
CFL2
SMIM1
ALCAM
ZNF217
MID1
SMAD5
TEX30
VARS2
TM6SF2
KCND3
DEXI
FANCC
ATL1
ABCA6

GNAI1
ACTR1A
ADIPOR2
CAV2
PMM1
PAPPA
ITIH3
RHOD
SLC25A6
LOC100289230
NEBL
STYX
ANXA9
CNTLN
C16orf80
ATP6V1G1
NUDT16
PRKAG2
NAA20
VOPP1
PLD1
RP1-30M3.5
ATP6V0B
CBR4
ACBD5
PLXNC1
FCGRT
TANGO6
OSBPL3
ACAT1
PLA2G4A
ILK
FAM83G
CD4
FAM162A
PITPNM3
HPSE
IKZF5
HEG1
DDOST
SLC41A2
CARD16
NR1H3

ORC2
ACADL
TMEM184B
MAGED2
RALA
TRAM1
THNSL2
PRPS1
MBD4
STAMBPL1
TMEM140
FIG4
ZNF652
FAM213A
DYNC1I2
VKORC1L1
TIMM9
GNPAT
SRA1
PFKM
KIAA1147
ARHGAP18
GUCY1A3
OST4
PECAM1
EDIL3
SLC35A3
PPP6R2
SRM
RP11-740C1.2
PGRMC2
PMM2
CCT6B
CAPN2
IVD
CASP4
F7
TMEM185B
A1CF
KIAA0922
DNAJC19
ALDOB
SEC24B

FADD
RHBG
RP1-86D1.3
CDK2AP1
EHD3
ADAM10
GAL3ST4
PKM
FKBP9
TRAPPC1
IL10RB
RBP5
PPARA
GLRX
ZNF195
ARG1
FZD5
MORC3
BTBD10
RITA1
POLD4
YAP1
GJB1
LINC00324
NOP10
ITPR2
DHTKD1
MASP1
ARL6IP5
SNRPD2
PAXIP1-AS1
BCL2L1
KAT2B
SLC29A1
JKAMP
TTC9
RP1-193H18.2
LRP6
SLC25A10
BPHL
NUDT13
RIT1
EXOC3L4

PIPOX
TPPP2
MOGAT3
PECR
GPD1L
PROC
LARP6
SMARCA5
TMSB4X
PBDC1
VASP
MSANTD3
ATP6V1B2
FUOM
ADCK1
C11orf54
FBXL4
ZNFX1
BC062753
TP53
PID1
NAP1L1
PTOV1
CD320
NOL3
CEBPG
NPAS2
CDC42SE1
TBCB
BRCA1
PDE7B
HSP90AA1
MYH9
MAMLD1
ZBTB48
SERPINB8
GLRX5
SEC31A
MICU3
KIAA0930
DAB1
CXorf38
ZCCHC17

GPM6A
PNP
NAPRT
ASNA1
11-Sep
GRB14
GPR137B
MOCS2
1-Mar
STIP1
GPC3
GPR56
C3orf14
PKDCC
TMEM243
TMTC1
FAM50A
CRYBG3
MAP3K1
COL9A3
NRTN
PARP4
PFN1
AP2B1
LRRC16B
SLC10A1
AGPAT5
ISOC2
GNAO1
PSME3
LOC339803
DAAM2
TMEM44-AS1
TMEM181
CES5A
CCDC113
LBX2-AS1
ADAMTS13
LIMA1
ITGB5
VIMP
FAM65C
GBAS

FAM46C
PRKAB1
SLC2A12
SAT2
SGCB
SLC25A1
RTCB
SLC16A7
TLR5
MFHAS1
ESYT1
RRAS
CCDC107
BHMT2
HADH
EMP1
TFR2
OTUD6B
WDR13
MFSD5
SLC30A10
DNAJB6
AMN
AMD1
CKAP4
SERPINB1
NOL8
PTPRB
ZNF281
CTSF
PEX11G
FADS3
SUGCT
C6
HAO1
IKBKG
TMED9
MUC20
ZUFSP
ACTN4
ITCH
SQRDL
PDK2

TJP1
CPOX
EPB41L5
PTTG1IP
CNRIP1
MST1L
TCEAL8
ACSL1
RGS3
LPAL2
ARPC2
GCLC
ENKD1
DRAM1
RAB17
IGHM
POLD2
RAB33B
RASSF3
PDE1A
ACSL5
ATP6V0E2
CAPNS1
HNRNPAB
COL4A4
MDN1
C1orf168
ACAA1
TWSG1
TXNRD1
PRKCDBP
MAOA
ANKRD55
ABAT
NEU1
DDX39A
IL17RC
HSBP1L1
BDH2
TAB2
MYO5C
LSS
PAK1IP1

PPP1R10
STS
TKT
DBT
CDKN2B
HIGD1A
SLC29A3
GNL2
CHN2
ARMC6
CITED2
RANBP3L
GSR
SEMA5A
SHC1
ACOT13
UBAP2
ZNF823
CASC10
SPAG1
LOC441124
ANXA2P3
CD63
C9
GAMT
KIAA1598
DRAM2
TDRD6
ABCC9
C15orf52
ASPSCR1
RP13-270P17.3
RBMXL1
FAM221A
LAMP2
PPAPDC1A
CAT
ST14
CYP2C18
CDC37L1
TUBE1
CYB5D2
CENPC

SHROOM2
LDLRAD3
RARRES2
TUFT1
ZBTB10
RASEF
E2F3
SERPIND1
HLA-B
GGTLC1
PPP1R3B
RPS27L
SLC22A7
PLOD3
ECM2
H1FX
ERO1L
ADAMTSL3
LIF
PHLDA2
TRIM21
PHF23
ADH1B
SCARNA17
NEDD4L
PRSS23
LCMT1
FERMT2
IQCH-AS1
RIC8B
PIGR
CARD6
TMEM55A
MBNL3
PPAP2A
B3GNT5
ASMTL
TMEM246
AMPD3
GOLGA2P5
CMTM8
PCBD1
TMEM256

ABHD17C
FAM127A
ADK
GCGR
NFIA
RP11-48B3.4
NARS
WIPI1
RP11-21L23.2
ARPC3
IFNGR1
LOC100287497
NET1
TESK2
MTHFD1
ALDH5A1
RMND5A
SEPN1
PALLD
LRRN3
C16orf87
SFN
DPH3
OCEL1
PPP1R14B
GCA
QPCT
FAM149A
ITIH1
CCT2
STARD3NL
TSPAN15
FGD4
MYCL
TCTEX1D2
VAMP5
PHYH
SESTD1
HSP90AB1
ATP6V1E1
HES4
ACBD4
TUBB

DPP3
PYCARD
C14orf169
SET
HLX
TIGD2
VAMP8
RAN
LOC100288675
KDR
SS18L1
RPP40
LIMK2
SCARA5
TMEM50A
B3GAT1
RHOQ
KIAA0485
ERO1LB
TRAM2-AS1
LRIG3
TRIB1
ARID4A
QDPR
TMEM2
CKLF
RBL2
ADRA1A
RCC2
LAIR1
PPA1
GRAMD1C
AQP9
WISP1
RAD54L2
MICU1
PRODH2
PPM1K
TSPYL2
LOC101927809
LASP1
LIFR
VWA5A

RFNG
PRR34-AS1
SLC18B1
COMMD8
AIFM2
CCND1
AAED1
CENPV
TMEM52
CD58
CYYR1
FAM127B
MYOF
LOC100131262
GALK1
TSPYL4
TSTA3
CAP1
BLOC1S2
MAP2
VGLL4
LOC100506990
RP4-758J24.5
HSD17B2
LDHD
GRHPR
MTFP1
PRSS53
HSD17B7
SLC37A4
HAPLN4
BMP2
LOC100289098
ZKSCAN1
ENDOD1
CCDC69
PLG
LINC01003
CCDC109B
DBP
PLK2
GCKR
MGMT

PGM1
ARL2
PCSK5
ALKBH2
NUBPL
SLC2A4RG
DTX4
NOP2
SCML1
CAPN5
SLC25A13
SMIM14
IL10
SELO
LHPP
MREG
ZNF511
PDLIM1
EBLN2
TES
ICAM3
CSTB
FNDC4
TMEM120A
SERTAD2
FRY
AIG1
CTD-2325A15.5
TPM4
SMO
ST3GAL6
TTR
PPP4R1
DNAJC6
PKD2
RPS6KA1
LOC101928076
HMGN1
IGLL5
SLITRK3
MAP1LC3A
EFCAB7
FGL1

CPD
SEL1L3
ID3
NADK2
CORO1C
FAM76B
TGM2
HSPB1
FAM214A
REPS1
ECSCR
TMCO3
BOK
MICAL1
PAPSS1
HRAS
RAB11B-AS1
IKBIP
TMEM54
GRAMD3
ABRACL
IYD
NFKBIE
C4orf19
CES3
RP11-384L8.1
GPR126
ATP11C
RALGPS2
CTR9
C11orf71
PAN2
NAAA
RRS1
MAGI2-AS3
SLC22A25
TCP10L
FBXO25
ZRANB1
CNST
GSKIP
TSPAN33
FUCA2

MMACHC
BSG
USP30
MYO6
C3P1
PCK1
CBS
RBBP8
SULF1
CXCL5
CTSD
ASPN
TMEM170B
WDR72
KLB
NDRG2
LPGAT1
ADCY1
PON3
SERINC5
GORAB
MYO1B
WSB2
KIAA1522
FMO3
BAX
ZNF275
FAM102B
PPAP2B
SDC2
HAGH
UNC93A
TACSTD2
LOC100507535
HEY2
ACLY
INHBC
ATP10D
B4GALT5
C7orf49
GPHN
DLAT
PON2

SLFN12
C21orf91
ACSM5
PANK1
PARP12
PEMT
COBLL1
NTF3
SEC14L4
PHYHD1
RAB37
CNNM3
THNSL1
N4BP2L1
LEPROTL1
FAM60A
PIK3R1
TM4SF1
IGF1
EBP
IL17RB
ARMCX2
TMEM87B
ENHO
DYNLL1
KLF15
SHF
SH3BGRL2
GPER1
ADORA2A-AS1
ALDH7A1
NREP
LRRC1
PPIF
GTF3C6
DNAJC12
GYG1
OGDHL
ZNF680
GJA1
CS
CTSC
TNFAIP8

SPATA20
TRIM6
C7orf55
TNFSF10
SERPINF2
TXN
MAMDC4
MYL12B
ACOX2
P2RX4
SLC27A4
GMFB
FICD
ZNF267
EPB41L4B
ZMYND12
SQSTM1
GYS2
SLC27A5
CTSA
ZNRF3
CMBL
SLCO2A1
TNFAIP8L1
DNASE1L3
RAB11FIP1
CA2
MSMO1
TCEB3-AS1
OSMR
TUBA1B
ALDH2
HGF
RP11-96D1.11
HMGB3
NEU4
NCOR1
COX7A1
VWF
LINC00261
EXPH5
PTGFR
USP18

PCK2
SIAH2
SERPINA4
UGCG
ACADS
TESC
PTH1R
BAMBI
RAMP3
HPR
SUCO
KBTBD11
ENPP1
SC5D
TPM1
SLC39A5
ALAD
ACOT9
PFKP
PVRL3
ENPP3
CYP27A1
AGL
HN1
ZCCHC6
PNMA1
AZGP1
CPED1
ENO3
DYNLT1
BCKDHB
SLC39A10
GIPC2
S100A10
TLR1
TMEM56
VNN1
FAM171A1
HRSP12
ANXA10
SUSD2
NTHL1
MANF

ATP1A1
BAAT
HSD17B6
ALDOA
BEX4
RNASE1
AOX1
NR1I3
SALL1
RNASE2
ATF7IP2
CYP1B1
FAM198A
CNGA1
FAM213B
FYN
PBLD
SLCO1B1
BAG3
UAP1
TNFRSF11B
CYP2C9
FSTL1
DEPDC7
MOGAT2
GARS
MPDZ
CHMP4C
SLC25A33
ADI1
ACVR1C
KLKB1
LOC100653086
TSPO
TMPRSS6
LDLR
SLCO4C1
TUBB3
SORT1
PPDPF
IFNGR2
GAS2L3
AMT

MUT
DBNDD1
SH3BGRL3
CYP4F3
SERPINE2
KCNJ8
ATP8B4
ASNS
GPT
ARRDC2
SMOC1
FAXDC2
GLUD1
OSBPL6
BCAT1
ST3GAL1
USP43
HFE2
ASPDH
ADHFE1
SELENBP1
ZMAT3
SACS
ARPC1B
CTPS1
CYP4F2
PDGFA
ANKRD37
ACSM2A
SLC17A2
TCTEX1D1
PLTP
N4BP2
FAM126B
CHST7
TUBB4B
GFRA1
RPL22L1
MFSD6
TMEM27
GCHFR
TMCO6
SLC25A18

DLEU1
NR1I2
EBPL
MPND
CCNB1IP1
LOC102723864
SLC13A5
SLC26A2
ASPA
LRRK2
CTSK
GAS2
LIPC
PXMP2
HMGCS2
RCAN1
MARCKS
RUNDC3B
CD151
ASPG
DCAF11
LOXL1
IGFBP1
EPHX2
CYSTM1
MOSPD1
LOC728040
TRNP1
RELN
PODXL
FRMD6
PON1
TOB1
RGN
ACKR3
WLS
ARSJ
ACAT2
MOGAT1
RAB8B
DPYS
MAT1A
LCAT

PLOD2
AGXT
GPX3
ABCB4
RP5-1092A3.4
SSTR1
NR5A2
LOC101927287
PLCXD3
GPLD1
C6orf123
CD109
CRY2
LIPG
TMSB10
CYP4A11
CYP2A6
HPGD
FAM117A
FLNA
RP11-355B11.2
ROBO1
SLC38A1
ODC1
STAG3
TUBA4A
LGALS1
APOF
GCAT
TUBB2A
RP4-680D5.8
NECAB2
ABCC6P1
SOX4
KLHL15
ITGBL1
C4orf48
HEXB
SIGIRR
AQP11
AVPI1
PKIB
IRS1

PPIC
LNP1
TMEM45A
DHRS1
NR3C2
CFHR5
AR
LGALS3
SULF2
ACSM2B
SPSB1
TAT
AZGP1P1
ANO1
FBLN5
TRIM47
IGFBP3
RNASE6
CDKN1A
MCAM
ALDH1L1
AP000253.1
SPINT1
PCOLCE
DSG1
MAN1C1
ALDH6A1
TUBB6
TUBA1C
HLF
UBE2Q2
PF4V1
ERMP1
DSG2
TOMM40L
SERPINA5
RP1-151F17.2
HOGA1
HGD
ELF3
LOC101928505
TAGLN2
CHAD

ADH1A
TSLP
CCDC146
SGK223
MAP2K1
GLYCTK
SLPI
ACMSD
F13B
IFIT2
LOC157273
APOA5
SELM
CYP2A7
LAMB1
NPY1R
SERPINH1
CA5A
KANK4
LGALS3BP
MVP
PNPLA3
SPA17
PRRG4
ACACB
DBH-AS1
SLC16A2
SRD5A1
EP300-AS1
PALM2
KHK
MLXIPL
HMGCS1
GCDH
SEC14L2
GNE
DMGDH
GSTP1
CXCL2
CDHR5
MPV17
TUBA1A
CH25H

SULT1C2
FCN2
ABHD15
PAIP2B
TTPAL
CYP3A5
LBP
INHBE
SLC12A8
TM7SF2
DFNA5
PRSS3
THOP1
WBP5
ARHGEF26
GLS2
CA12
CYP2C8
LOC730101
PKLR
ABCG2
C7
SKAP1
SORL1
MROH2A
FXYD1
FTCD
STARD4
DGAT2
CYP4F12
LOC100130232
CDO1
SORD
TP53INP2
CYP3A43
UPB1
RARRES1
FZD6
RCL1
ADH1C
SLC47A1
LIME1
DCDC2

ISG20
GPX2
HIST1H2AC
PRG4
DUSP10
CYP39A1
LOC101929475
DNMT3L
TGFBI
TMEM139
DCXR
ETNPPL
HGFAC
SLC51A
TGDS
CXCL14
CRYAB
G0S2
RP11-250B2.6
CYP4X1
CLDN14
ACADSB
SRXN1
COQ10A
FABP4
CFP
PSAT1
GSTZ1
PLIN1
GBA3
IL6R
ANXA5
CLDN7
FMO5
KPNA2
ADH4
SLC38A4
HSPA2
PTGFRN
CUX2
C12orf5
FITM1
IL1RAP

GPR125
KRT8
TSPAN7
LOC101926960
HTR2B
GSTT2
KRT18
DAK
LOXL4
PHGDH
S100A11
IER3
FKBP1B
GSTA3
RAB26
RAPH1
SLC22A1
PROZ
HAAO
CRP
SLC22A15
BACE2
ADRB2
CAP2
FETUB
SLC6A1
LOC100505985
ADH6
ASB9
VSNL1
LOC149703
CIDEC
IGFALS
CLDN2
FAM169A
ANXA2
GPR128
EPDR1
VIPR1
TP53I3
ERICH5
FOLH1B
PCDH17

GPR34
IGJ
BEX2
MLLT11
FGF13
SCG5
ZNF385B
ENPP2
RDH16
SLC16A10
FLJ22763
SLC35C1
GLYAT
INSIG1
G6PC
PGLYRP2
LYVE1
CHI3L1
CTH
LOC100507389
DHRS2
TTC36
FAM151A
LOC101927331
EGR1
PLP2
PDK4
AADAT
CETP
DEFB1
ACSM3
CLEC4G
SULT1E1
LOC102723845
TIMP1
TNFRSF12A
ZGPAT
MME
AASS
LGALS4
IL13RA2
SLC44A3
ANXA13

ETNK2
BCHE
ANXA2P2
SOX9
C5orf27
FAT1
HAO2
PPP1R1A
ZG16
THRSP
CLEC4M
EEF1A2
CPS1-IT1
CFHR4
GPR88
CFHR3
SRD5A2
SLCO1B3
VCAN
STMN2
NQO1
IL32
SPP1
LPA
AKR1D1
LINC00844
C15orf48
CNDP1
CXCL1
CD5L
CD24
BBOX1
SLC51B
CYP2C19
CXCL8
PCOLCE2
LINC01093
S100P
CYP7A1
GNMT
CXCL6
CYP1A2
LCN2

KCNN2
CCL20
KRT23
