# Supplementary material for: Role of MIF in coordinated expression of hepatic chemokines in patients with alcohol-associated hepatitis
Source: JCI Insight. 2021 Jun 8;6(11):e141420. doi: 10.1172/jci.insight.141420 (PMC8262327; doi:10.1172/jci.insight.141420)
Supplement: Supplemental Table 2 [file jciinsight-6-141420-s153.pdf]

TMEM37
WDR41
AC068039.4
EIF2AK4
SIAH1
ANKRD17
MINA
HAT1
CMSS1
CHRD
FASTKD3
SEL1L
EPB41L4A-AS1
TMCO1
NUP93
PTRHD1
GHITM
ZNHIT3
HIVEP1
RABGGTB
SCAMP2
ARPC1A
ALG6
INTS7
CNBP
AHSA1
KLHDC7A
SLC25A32
COG5
GGPS1
TCF7L2
LZTS3
TIPRL
XPR1
H6PD
TLR3
ORC3
C1QBP
PELI2
TMEM260
NDUFA13
VPS25
BTG1

NIPA2
COPZ1
NIPSNAP3A
TJP2
PA2G4
CA5BP1
ENTPD7
RPL26L1
MED4
SCAF4
SKIL
NAF1
NDUFA1
ZNF35
TSHZ1
SLC30A7
MRPL1
CTDSP1
MTUS1
C14orf105
IGBP1
RALGAPA2
SMIM20
CPSF3
ARL1
DUSP3
MANBA
CCDC53
GEMIN2
TMEM167A
NIPA1
LINC-PINT
TSEN15
MRPL15
NDUFA8
TRIM25
PGK1
MYO5B
SRSF11
C1orf122
ACTL6A
ASUN
TM9SF2

MTPAP
USP32
XRCC4
ETFA
C6orf211
THTPA
ZCCHC7
COMMD9
EMP2
C9orf64
RTN4IP1
COA7
SKI
DRG1
PSMA1
AAAS
RAD23A
TIMMDC1
OIP5-AS1
FTSJ3
CLN3
AF007147
DSERG1
EDEM1
MSL2
S1PR1
SEC23A
PSMB5
LDLRAD4
SPCS1
SAE1
CSE1L
OTUD1
TBPL1
FTSJ2
HPRT1
KIAA1191
AGPAT1
ENO1
SNRPD3
NDUFS2
NFX1
BBS4

BRAF
MFAP1
SASH1
SHQ1
FKSG49
EIF2B4
BRF2
TAF12
MTERF1
GLRX2
KCNAB1
RP11-326I11.3
BRI3BP
FBXO2
CDKN1B
SPCS3
IFI27L1
SCAMP3
CCDC51
KCTD6
EDEM3
NDEL1
DLL1
PIGC
LGALS2
ALYREF
ACSL3
MLH1
R3HDM2
MPHOSPH6
LRRC8D
SLC39A14
CANX
POLR2K
RPAP3
MCAT
NAE1
PSMD7
GOLIM4
SCYL3
NDUFAF2
MOB3B
POP5

RLF
MGC12488
COMMD3
TMX1
TNPO1
PXDC1
ITGAD
UFC1
TMEM150C
TIAM1
RPL36A
VRK2
CDK8
INTS12
ANKRD50
PSMA7
SCOC
MEA1
PRDX1
EXT2
YWHAE
TOR1AIP1
IMMT
ZC3H13
TIGD1
TPRKB
GGCT
SSR1
NUDT9
EMG1
AACS
IVNS1ABP
PRDX4
LOC101927609
TMOD3
MANBAL
UPF2
DEDD2
PARP1
TTC37
GABPB1-AS1
PSENEN
RUSC1

KLF2
PRKAG1
ANAPC16
FBXW4
NAA10
ATL3
PLRG1
OGFOD1
NAA35
BRCC3
SSR3
CALR
CMTM4
PRKRIR
CDADC1
KATNA1
UPF3B
PDSS1
TLCD1
C6orf203
GINS2
TCTN3
PLSCR4
XRN2
IBTK
PSMC6
DNAJC10
EPAS1
SEC24C
RP3-368A4.6
UFD1L
EVL
BBX
MECR
YIPF3
PSD4
LOC340085
FN1
PSMA4
UCHL3
CDC123
PUS3
KIF16B

CLCN3
RPS6KC1
TCF7L1
ADSL
CYB561A3
TMEM106C
APBA1
TAF13
FIBP
CEACAM1
DLC1
KIF2A
XPNPEP1
ARF1
APRT
UBLCP1
SAAL1
SLC30A9
OCLN
PHF10
SLC35B1
SF3B5
PSMD10
CTC-425F1.4
JUP
RREB1
MRPL27
ARL4D
ABCE1
GRHL1
SETD1B
MACROD1
MYO9A
USP54
FOCAD
TMX2
FAM46A
ZNF512B
PDCD2L
TOB2
SNX29P2
EEF1E1
FDX1L

MUM1
SPRY2
ATP5G1
DAPK1
PMVK
MKKS
PRRC1
HTATIP2
XRCC6BP1
UQCRQ
PSMC4
UNC119
KAZN
GLMN
ITM2C
ASXL1
USO1
COX6B1
ABCA9
SLC25A43
NUDT19
FAM63A
TMEM198B
LACTB2
SATB2
ATP5B
AMFR
RANGRF
TBCK
TUSC1
PPP2R2A
TYW3
HSCB
WBP2
TMEM138
SCO2
SCARB2
CLMN
RP11-319G9.3
ILF2
HEATR5A
GPX4
C17orf75

RING1
CRYZ
NFKBIA
TXNDC12
HNRNPL
UBFD1
PDIA6
CFLAR
ALDH1A1
TXNL4B
HMOX2
FANCI
GINS3
LEO1
CKAP5
MRPS17
C1R
PSMD2
ENOSF1
GSS
HAUS1
CSK
MRPL17
NDUFA9
TMED2
EXOSC9
TMEM214
RPL39L
CDK10
PIGU
PNPT1
HPN
C1orf131
RPA2
MAD2L1BP
HMBS
FASTKD5
RNF138
CTDSP2
ADRB1
ALG3
C11orf73
DCK

ZBTB33
RRN3
NABP2
PLCXD1
NSMCE2
RP11-295G20.2
SNRK
PRMT3
LOC439911
CNPY3
ZNF322
ASCC3
DARS2
PIGF
AP3B1
ERF
ATR
NUP107
EXOC2
ARFIP1
LPIN2
DVL1
MRPL13
CCNL1
SSB
SKA2
P4HB
BC032415
STMN1
FHL1
MPZL2
SRP19
GADD45A
MLEC
RBMS1
USP32P2
CD82
NDUFA6
KPNA3
ANKRD39
ZDHHC2
ECT2
LOC100506100

RHOBTB3
TTC1
DENND4B
ZFP36L2
ATHL1
DPCD
SRPRB
KDM2B
SMC4
FAM107A
PJA1
PDZD11
CHPT1
TMEM92
RUVBL1
BPGM
FOXO3
IGFBP4
FUNDC1
SLC25A37
NR2C2AP
BARD1
PELI1
THG1L
FAM9B
TBC1D2
FAM20A
HSPA13
AKTIP
CTNNBIP1
APOO
SLIRP
MAST3
UBL7
SESN2
ZCCHC11
SEC61A1
TENC1
SLC22A3
ATG4A
BCL2L10
CLUH
EPRS

SOX7
ATIC
FURIN
DNAJB11
RRM1
PTER
FNBP1L
RRM2B
MAD2L2
RBM6
MLH3
ARL4A
PDE2A
KLHDC1
FUBP3
ZMPSTE24
HSPA4
RHOU
TRMT10C
IDE
RP11-769O8.3
ECD
SPSB3
IRF2BPL
TMEM258
SCO1
DUSP16
SNORD89
ZNF274
RPN2
PTRH2
AC012065.7
FANCG
MAN2A2
RORA
NDC1
SGOL2
MCM6
KCNJ3
SMC2
CACNB2
BRE
AGPS

GAPDH
RHOB
TOMM5
EIF2B2
TUBG1
DBF4
MXI1
SMAP2
UBXN8
GPI
RHNO1
PRKAA2
LONRF1
HS3ST3B1
GALNT1
GLT1D1
STT3A
ARHGEF10L
UXS1
PROX1
BCAP31
SLC35G1
RECQL
ING1
M6PR
ASB13
PPIH
BOLA3
YIPF1
ATXN7
PPARGC1A
C1RL
ACSS3
SOWAHC
POLR2G
KIAA1033
TMCC1
ZBED9
HARS2
XAF1
GOLGA8N
FKBP11
ARL2BP

PPAPDC1B
MCU
PLGRKT
NRM
CDC7
GOLT1B
FASTKD1
CETN2
MBOAT1
ALDH18A1
SNRNP40
COPG1
BCL6
SLC17A5
PGM3
FDXR
OPLAH
CTNNA3
SNX7
C2orf76
TMEM9B
ANKS1A
SIGLEC1
ZNF468
NEB
GFPT1
MDH1
BC022892
METTL18
HYLS1
AKAP9
LEAP2
THSD7A
CPEB4
ID2
RFX5
MLPH
ATG2A
SAC3D1
RXRA
VEGFA
GBAP1
FXYD6

CLIP4
FIGNL1
COL18A1
C8orf82
RCN2
AGA
STX18
CREB3
AADAC
XK
DAD1
TMEM117
PPIB
SLC38A2
ZFAND5
SEC61G
SPDL1
BRD1
DPAGT1
PRR18
LOC101928230
TMEM147
ME1
PSMD1
LRRC20
ASH1L-AS1
FEN1
HIST1H2BH
FAM134B
FAS
IARS
CD160
EOMES
ASPM
L3HYPDH
HYOU1
SPTSSA
C9orf72
KNSTRN
ITM2A
PPIL1
SUN2
DOLK

H2AFZ
AKAP12
ARHGAP10
CTB-50L17.7
TMEM220
SYPL1
ELOVL2
ARF4
KLF9
CDA
CLASRP
ULK1
TIPARP
HIST1H2BE
ADAMTS1
CCDC23
VRK1
CCT6A
PROCR
RTP3
MT1HL1
MCC
C4orf29
SLC26A6
TGFBR3
ACSL4
FOXO1
HIST1H2BK
GSAP
PCNA
PIR
TM4SF4
ZWILCH
FBXO6
PACSIN3
H2BFS
MSH2
GCH1
H2AFY2
RTP4
PAPD7
APOL3
DHRS7B

PPAT
HNRNPU-AS1
MAD2L1
CEBPB
ZDHHC23
IL1RN
JUN
CRNDE
MCM2
SPAG5
GPX7
PER2
GLA
KLRF1
ELL2
MIR3682
KIAA0101
RPS27
OVGP1
ZBED3
ENPP7
MT1E
NME1
TMEM98
CBX7
IL2RB
PSMD14
RP11-271C24.3
MND1
CKS2
LINC00238
HERC5
UHRF1
TSC22D3
MCM3
CCS
SGK1
SLC22A18
SLC6A16
NAMPT
RP11-297L17.2
ABCB1
FNIP2

ITGB3BP
TM4SF5
HSD17B14
HTRA1
ORMDL2
ATF3
TYMS
RFC4
MT1H
IRF8
MT1X
AJUBA
PHLDA1
DHODH
HAL
GPT2
PFKFB3
HPS5
CENPU
SMAGP
ANGPTL6
CDK1
TOP2A
FKBP5
UBE2C
SIRT1
AKR1C3
MT1G
GINS1
GMNN
IER2
ALAS1
HOMER2
KLF6
LOC101927972
HSPA4L
HIST1H2BC
NINJ2
AVPR1A
FAIM
ZFP36
ANO10
BUB1B

RPS16P5
ANLN
NCAPG
ITGA6
RORC
PLGLB2
UGT1A6
C8orf4
KRT222
RACGAP1
TPX2
JUNB
SOCS2
CISH
KDELR3
ERRFI1
RGS1
GOLM1
VMP1
CCNB1
NUSAP1
RAD51AP1
F2RL1
CENPW
DTL
SYBU
SLC1A2
IRS2
DDIT4
KIF20A
CDKN3
DUSP1
DIRAS3
CYP26A1
APOBEC3B
MORC4
GADD45B
HMMR
CHST9
NDC80
APOA4
MT1F
CAPN3

LEPR
CYR61
GPR64
ZBTB16
C19orf80
SLC19A2
SERPINE1
FST
AK025288
PRC1
EGR2
PBK
ZWINT
SLC25A25
CLGN
AOC1
RP11-138A9.1
LINC01018
CXCL10
EFHD1
TMEM45B
OAT
GADD45G
CYP1A1
PTTG1
LGSN
IDO2
SDS
PZP
RRM2
MT1M
FOSB
FOS
SPINK1
AKR1B10
