# Supplementary material for: Role of MIF in coordinated expression of hepatic chemokines in patients with alcohol-associated hepatitis
Source: JCI Insight. 2021 Jun 8;6(11):e141420. doi: 10.1172/jci.insight.141420 (PMC8262327; doi:10.1172/jci.insight.141420)
Supplement: Supplemental Table 5 [file jciinsight-6-141420-s156.pdf]

Gene	Forward (5’ to 3’)	Reverse (5’ to 3’)
sXbp-1	GAG TCC GCA GCA GGT G	GTG TCA GAG TCC ATG GGA
Grp78	ACT TGG GGA CCA CCT ATT CCT	ATC GCC AAT CAG ACG CTC C
Chop	CTG GAA GCC TGG TAT GAG GAT	CAG GGT CAA GAG TAG TGA AGG T
Dr5	GGT CCT CTT GAT GGG CTC TC	GTT GCT GCT TGC TGT GCT AC
Cxcl1	TGC ACC CAA ACC GAA GTC	GTC AGA AGC CAG CGT TCA CC
Lix	TCC TCA GTC ATA GCC GCA AC	GCT TTC TTT TTG TCA CTG CCC A
Ccl2	AGG TCC CTG TCA TGC TTC TG	TCT GGA CCC ATT CCT TCT TG
Ccl20	ACT GTT GCC TCT CGT ACA TAC A	GAG GAG GTT CAC AGC CCT TTT
Ly6g	TGC GTT GCT CTG GAG ATA GA	CAG AGT AGT GGG GCA GAT GG
Cxcr2	ATG CCC TCT ATT CTG CCA GAT	GTG CTC CGG TTG TAT AAG ATG AC
Ccr2	AGG AGC CAT ACC TGT AAA TGC	TAG TCA TAC GGT GTG GTG GC
18s	ACG GAA GGG CAC CAC CAG GA	CAC CAC CAC CCA CGG AAT CG
